# The association between obesity and weight loss after bariatric surgery on the vaginal microbiota

**DOI:** 10.1186/s40168-021-01011-2

**Published:** 2021-05-28

**Authors:** Olivia Raglan, David A. MacIntyre, Anita Mitra, Yun S. Lee, Ann Smith, Nada Assi, Jaya Nautiyal, Sanjay Purkayastha, Marc J. Gunter, Hani Gabra, Julian R. Marchesi, Phillip R. Bennett, Maria Kyrgiou

**Affiliations:** 1 IRDB, Department of Metabolism, Digestion and Reproduction - Surgery and Cancer, Hammersmith Campus, Imperial College London, W12 0NN London, UK; 2 Queen Charlotte’s and Chelsea-Hammersmith Hospital, Imperial College NHS Trust, W12 OHS London, UK; 3 March of Dimes European Prematurity Research Centre, Imperial College London, London, W12 0NN UK; 4 Division of Population Medicine, Cardiff University, Heath Park, Cardiff, CF14 4YS UK; 5 Section of Nutrition and Metabolism, International Agency for Research on Cancer (IARC), 150 Cours Albert Thomas, Lyon, France; 6 St Mary’s Hospital, Imperial College NHS Trust, W2 1NY London, UK; 7 Division of Integrative Systems Medicine and Digestive Disease, St. Mary’s Hospital, Imperial College London, South Wharf Road, London, W2 1NY UK

**Keywords:** Obesity, Overweight, Body mass index, BMI, Vaginal microbiota, Bariatric surgery

## Abstract

**Background:**

Obesity and vaginal microbiome (VMB) dysbiosis are each risk factors for adverse reproductive and oncological health outcomes in women. Here, we investigated the relationship between obesity, vaginal bacterial composition, local inflammation and bariatric surgery.

**Methods:**

Vaginal bacterial composition assessed by high-throughput sequencing of bacterial 16S rRNA genes and local cytokine levels measured using a multiplexed Magnetic Luminex Screening Assay were compared between 67 obese and 42 non-obese women. We further assessed temporal changes in the microbiota and cytokines in a subset of 27 women who underwent bariatric surgery.

**Results:**

The bacterial component of the vaginal microbiota in obese women was characterised by a lower prevalence of a *Lactobacillus*-dominant VMB and higher prevalence of a high diversity (*Lactobacillus* spp., and *Gardnerella*- spp. depleted) VMB, compared with non-obese subjects (*p*<0.001). Obese women had higher relative abundance of *Dialister* species (*p*<0.001), *Anaerococcus vaginalis* (*p*=0.021), and *Prevotella timonensis* (*p*=0.020) and decreased relative abundance of *Lactobacillus crispatus* (*p*=0.014). Local vaginal IL-1β, IL-4, IL-6, IL-8, IFNγ, MIP-1α and TNFα levels were all higher among obese women, however, only IL-1β and IL-8 correlated with VMB species diversity. In a subset of obese women undergoing bariatric surgery, there were no significant overall differences in VMB following surgery; however, 75% of these women remained obese at 6 months. Prior to surgery, there was no relationship between body mass index (BMI) and VMB structure; however, post-surgery women with a *Lactobacillus*-dominant VMB had a significantly lower BMI than those with a high diversity VMB.

**Conclusions:**

Obese women have a significantly different vaginal microbiota composition with increased levels of local inflammation compared to non-obese women. Bariatric surgery does not change the VMB; however, those with the greatest weight loss 6-month post-surgery are most likely to have a *Lactobacillus*-dominant VMB.

**Video abstract**

**Supplementary Information:**

The online version contains supplementary material available at 10.1186/s40168-021-01011-2.

## Background

Obesity has become a worldwide problem, with projections of 1 in 5 women being obese and 1 in 10 morbidly obese by 2025 [1]. The gynaecological complications of obesity include menstrual disorders [2, 3], anovulation [4], polycystic ovarian syndrome [5, 6], infertility [7–9], early pregnancy loss [10–12], preterm birth [13], obstetric complications [13, 14] and increased risk for gynaecological malignancies (endometrial and ovarian) [15–18]. The mechanism by which obesity promotes several of these outcomes is not fully understood.

There is growing recognition that microbiota (i.e. the assemblage of microorganisms found at a specific environment [19]) and their functions influence disease pathophysiology [20–22]. Shifts in microbiota composition at mucosal surfaces can lead to pathobiont overgrowth and activation of innate immune responses that in turn are modified by microbial products such as short-chain fatty acids, lipids, and bioamines [23–26]. The vaginal microbiota is commonly dominated by *Lactobacillus* spp. [27, 28], which offer protection against colonisation of pathogenic bacteria through production of lactic acid, lowering of vaginal pH, production of antimicrobial compounds and modulation of both the immunological and physical properties of cervicovaginal mucosa [29, 30]. High-throughput sequencing approaches have aided characterisation of the vaginal microbiota in health and disease states, leading to a better understanding of the factors which affect vaginal community composition including age and ethnicity [31], menstrual cycle phase [32], oestrogen levels and menopause status [33], intercourse [34], pregnancy [35, 36] and hygiene practices [37].

Bacterial vaginosis (BV), characterised by a shift from *Lactobacillus* spp. dominance towards high relative abundance of anaerobes, has long been associated with a range of adverse outcomes [38]. Molecular-based characterisation of vaginal bacteria composition has extended these findings to permit identification of specific taxa that modify risk of preterm birth [39–42] and preterm premature rupture of membranes [43], sexually transmitted infections [44], human papilloma virus and cervical disease [45–47].

Obesity and specific vaginal microbiome (VMB) compositions are each risk factors for adverse reproductive and oncological health outcomes in women, but there is limited evidence describing the relationship between vaginal microbiota composition and obesity and the impact that body weight may have on local inflammation, immune response and health outcomes. Brookheart and colleagues found that overweight and obese women have higher Nugent scores and a greater occurrence of BV [48]. Conversely using data from the US National Health and Nutrition Examination Survey 2001-2004, Koumans and co-workers concluded that body mass index (BMI) was not an independent risk factor for BV [49]. Two Korean studies have examined the relationship between VMB, assessed by metataxanomic analysis, and obesity. Oh and colleagues found that obesity associates with cervical microbiota dominated by *Lactobacillus iners* [50]. Si and co-workers reported that discordant twin obesity associated with increased bacterial diversity and prevalence of *Prevotella* [51].

In this study, we investigated how obesity, defined as BMI ≥30 kg/m^2^, associates with bacterial structure and composition of the vaginal microbiome compared to non-obese women and local inflammation. In a secondary analysis, we investigated the effects of bariatric surgery on vaginal bacterial diversity and local inflammation.

## Results

A total of 109 women were prospectively recruited into this study, 67 were non-obese and 42 were obese (Table 1). Non-obese women were more likely to be nulliparous (*p*<0.001), non-diabetic (*p*=0.001), using contraception (*p*=0.016) and Caucasian (*p*=0.024) compared to obese women. There was no difference in mean age (*p*=0.083), smoking (*p*=0.128) or menopause status (*p*=0.233) (Fisher’s exact test). No differences were found between recent antibiotic use (*p*=0.258) or time since last sexual intercourse prior to sample collection (*p*=0.070) (data not shown).

**Table 1:** Patient characteristics of the whole cohort at baseline sample collection (*n*=109) according to obesity status

Characteristics	Non-obese (***n***=67)	Obese (***n***=42)	Total (***n***=109)	***p*** value^**a**^
**Age (years)**				0.083
Mean (SD, range)	44 (11.79, 20-75)	46 (11.26, 28-72)	44 (11.79, 20-75)	
**Ethnicity,** ***n*/*N*** **(%)**				**0.024**
Caucasian	48/67 (71.6)	22/42 (52.4)	70/109 (64.2)	
Asian	3/67 (4.5)	5/42 (11.9)	8/109 (7.3)	
Black	12/67 (17.9)	15/42 (35.7)	27/109 (24.8)	
Other	4/67 (6.0)	0/42 (0)	4/109 (3.7)	
**Parity,** ***n*/*N*** **(%)**				**<0.001**
Nulliparous	44/67 (65.7)	7/42 (16.7)	51/109 (46.8)	
Parous	23/67 (34.3)	35/42 (83.3)	58/109 (53.2)	
**Smoking status,** ***n*/*N*** **(%)**				0.128
Current smoker	11/67 (16.4)	2/42 (4.8)	13/109 (11.9)	
Non-smoker	56/67 (83.6)	40/42 (95.2)	96/109 (88.1)	
**HVS results** ***n*/*N*** **(%)**				0.062
Normal	46/67 (68.7)	36/42 (85.7)	82/109 (75.2)	
Abnormal	15/67 (22.4)	6/42 (14.3)	21/109 (19.3)	
Unknown	6/67 (8.9)	0/42 (0)	6/109 (5.5)	
**Abnormal HVS results,** ***n*/*N*** **(%)**				0.076
Bacterial vaginosis	3/15 (20.0)	1/6 (16.7)	4/21 (19.0)	
*E. coli*	1/15 (6.7)	0/6 (0)	1/21 (4.8)	
*S. aureus*	1/15 (6.7)	0/6 (0)	1/21 (4.8)	
*Group B streptococcus (S. agalactiae)*	2/15 (13.3)	3/6 (50.0)	5/21 (23.8)	
Yeast	0/15 (0)	1/6 (16.7)	1/21 (4.8)	
Mixed coliforms	0/15 (0)	1/6 (16.6)	1/21 (4.8)	
Unknown	8/15 (53.3)	0/6 (0)	8/21 (38.0)	
**Menopause status,** ***n*/*N*** **(%)**				0.233
Premenopausal	50/67 (74.6)	26/42 (61.9)	76/109 (70.0)	
Postmenopausal	17/67 (25.4)	16/42 (38.1)	33/109 (30.0)	
**Phase of menstrual cycle (PrMP),** ***n*/*N*** **(%)**				0.115
Luteal	24/50 (48.0)	7/26 (26.9)	31/76 (40.8)	
Follicular	15/50 (30.0)	8/26 (30.8)	23/76 (30.3)	
Ovulation	0/50 (0)	1/26 (3.8)	1/76 (1.3)	
Unknown	11/50 (22.0)	10/26 (38.5)	21/76 (27.6)	
**Use of contraception (PrMP),** ***n*/*N*** **(%)**				**0.016**
Nil	27/50 (54.0)	20/26 (77.0)	47/76 (61.9)	
Condoms	5/50 (10.0)	0/26 (0)	5/76 (6.6)	
COCP	12/50 (24.0)	1/26 (3.8)	13/76 (17.1)	
POP	1/50 (2.0)	0/26 (0)	1/76 (1.3)	
Copper IUD	0/50 (0)	0/26 (0)	0/76 (0)	
Mirena IUS	3/50 (6.0)	5/26 (19.2)	8/76 (10.5)	
Vaginal ring	0/50 (0)	0/26 (0)	0/76 (0)	
Contraceptive implant	2/50 (4.0)	0/26 (0)	2/76 (2.6)	
Contraceptive injection	0/50 (0)	0/26 (0)	0/76 (0)	
**Use of HRT (PoMP),** ***n*/*N*** **(%)**				0.175
Yes	5/17 (29.4)	1/16 (6.2)	6/33 (18.2)	
No	12/17 (70.6)	15/16 (93.8)	27/33 (81.8)	
**Diabetes status,** ***n*/*N*** **(%)**				**0.001**
Non-diabetic	65/67 (97.0)	32/42 (76.2)	97/109 (89.0)	
Diabetic	2/67 (3.0)	10/42 (23.8)	12/109 (11.0)	
**Diabetic treatment,** ***n*/*N*** **(%)**				0.212
Diet control only	1/2 (50.0)	3/10 (30.0)	4/12 (33.3)	
Metformin alone	0/2 (0)	3/10 (30.0)	3/12 (25.0)	
Metformin combined 2nd diabetic medication	0/2 (0)	0/10 (0)	0/12 (0)	
Other oral antiglycaemic medication	1/2 (50.0)	0/10 (0)	1/12 (8.4)	
Insulin (alone or with oral medication)	0/2 (0)	4/10 (40.0)	4/12 (33.3)	
**HOMA-IR**^**b**^**,** ***n*/*N*** **(%)**				0.136
Insulin resistant	2/67 (3.0)	5/42 (11.9)	7/109 (6.4)	
Non-insulin resistant	5/67 (7.5)	5/42 (11.9)	10/109 (9.2)	
Unknown insulin resistance status	60/67 (89.5)	32/42 (76.2)	92/109 (84.4)	

*BMI* body mass index, *COCP* combined oral contraceptive pill, *E. coli Escherichia coli*, *HOMA-IR* homeostatic model of assessment-insulin resistance, *HVS* high vaginal swab, *IUD* intrauterine device, *IUS* intrauterine system, *PoMP* postmenopausal, *POP* progesterone-only pill, *PrMP* premenopausal, *S. aureus Staphylococcus aureus*, *SD* standard deviation

^a^Calculated using Fisher’s exact test. A *p* value of less than 0.05 demonstrates a significant difference in the distribution of the demographic of interest (e.g. ethnicity), according to obesity status

^b^HOMA-IR was calculated according to the formula: fasting insulin (μU/L) multiplied by fasting glucose (nmol/L)/22.5. The 2nd tertile was used as the cut-off to determine insulin resistance status. Insulin resistance cut-off value, 2.98

A subset of twenty-seven of the 109 recruited women were scheduled for bariatric surgery and were sampled at baseline (*n*=27), 3 months (*n*=22) and 6 months (*n*=19) post-surgery. Cohort characteristics are shown in Supplementary Table 1. The mean age was 48 years (SD 8.91, range 28-65 years), and the majority of women in the bariatric surgery cohort were Caucasian (18/27, 66.7%), premenopausal (17/27, 63.0%) and only one postmenopausal participant used HRT. One third of the cohort were diabetic (8/27, 29.6%), and half of the women who were diabetic required insulin. We found that 44% of the cohort was insulin resistant at baseline.

In the 16S rRNA gene sequencing analysis, a total of 3,045,614 reads were captured from 166 samples (109 baseline, 57 longitudinal samples) with an average number of reads per sample of 18,021. To avoid sequencing bias, operational taxonomic units (OTUs) were randomly sub-sampled to the lowest common read count of 1885 with coverage of greater than 95% (Good’s coverage index) being maintained for all samples. A total of 265 taxa were detected in the study cohort microbiota after the removal of singletons and rare OTUs. Rare OTUs were defined as those present at less than 10 counts within the entire cohort, and along with singletons, were included in the final analysis to maintain consistent read counts across all samples.

### Vaginal microbiota composition and local cytokine expression according to obesity, diabetes and insulin resistance status

**Fig. 1:**
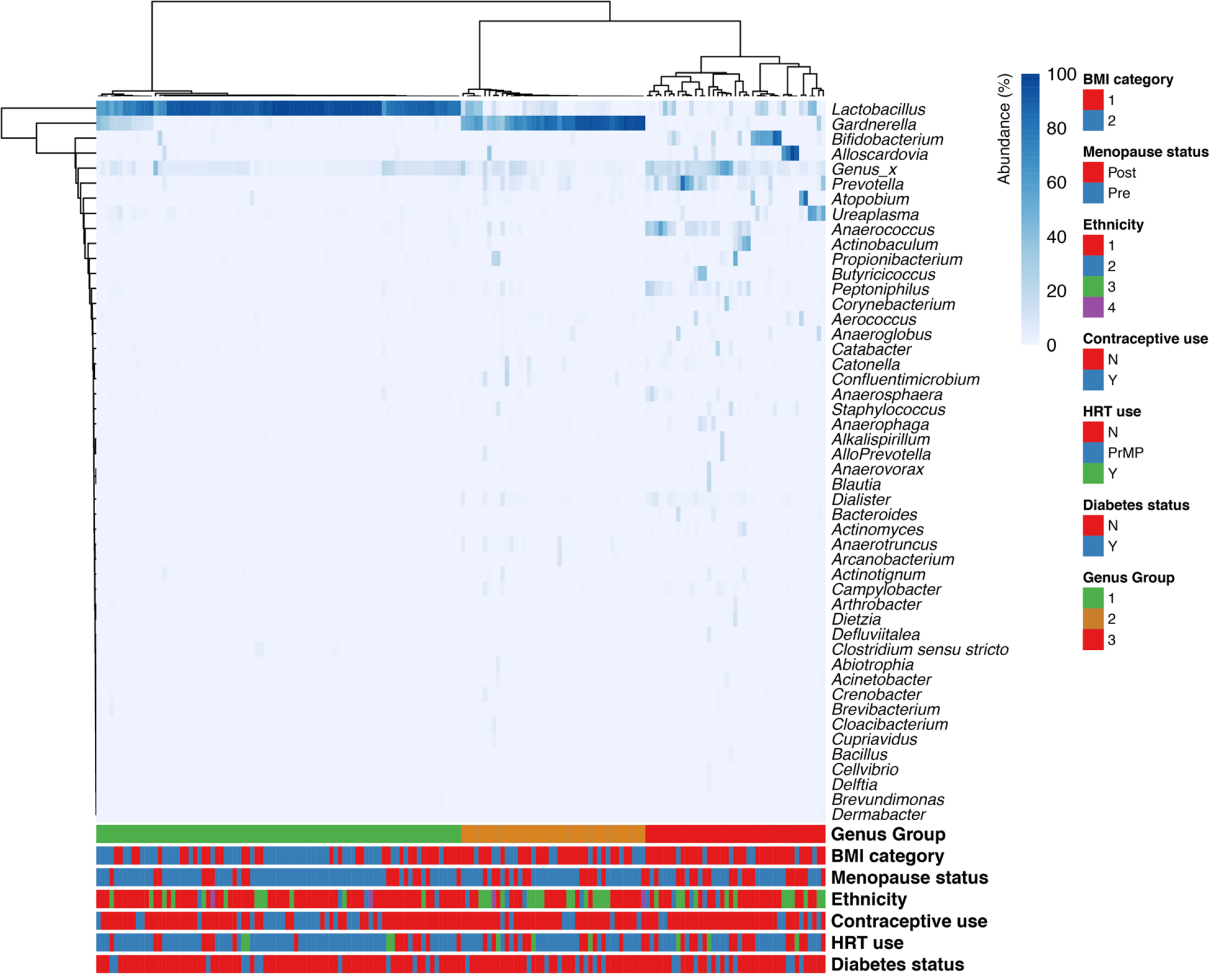
**a** Hierarchical clustering analysis of genera taxonomic level data generates three distinct groups consisting of (i) *Lactobacillus*-dominant (characterised by high relative abundance of *Lactobacillus* spp.), (ii) *Gardnerella*-dominant (characterised by high relative abundance of *Gardnerella* spp. and low relative abundance of *Lactobacillus* spp.) and (iii) high diversity vaginal microbiome (VMB)—characterised by low relative abundance of each of *Lactobacillus* and *Gardnerella* species, and increased bacterial diversity. Heatmap created from all samples collected (*n*=166), using Ward linkage with the fifty most commonly identified microbial genera shown. Cohort characteristics including BMI category, menopause status, ethnicity, contraceptive or hormone replacement therapy (HRT) use and diabetic status are also shown below the heatmap

Ward hierarchical clustering analysis of genera-level data identified three major groups, on the basis of relative bacterial abundance: (i) *Lactobacillus*-dominant—characterised by high relative abundance of *Lactobacillus* spp., (ii) *Gardnerella*-dominant*—*characterised by high relative abundance of *Gardnerella* spp. and low relative abundance of *Lactobacillus* species and (iii) high diversity VMB—characterised by low relative abundance of each of *Lactobacillus* and *Gardnerella* species, and increased bacterial diversity (Fig. 1a). Across the whole cohort, the prevalence of *Lactobacillus*-dominant and high diversity VMB groups were significantly different (Fig. 1b). The frequency of the bacterial groups within patient groups subcategorised on the basis of obesity status, diabetes and insulin resistance status is presented in Table 2. Prevalence of the high diversity VMB was significantly greater in obese women (obese; 18/42 (42.8%) compared to non-obese; 10/67 (14.9%), *p*=0.002) whilst the prevalence of *Lactobacillus*-dominant VMB was significantly lower (Table 2). There was no difference in the prevalence of *Gardnerella*-dominant VMB between obese and non-obese women. There were overall no significant associations between the three major vaginal bacterial groups and diabetes (Table 2) or where diabetic status was subcategorised into obese or non-obese women (Supplementary Table 2).

**Table 2:** Prevalence of genus group present according to obesity status, diabetes and insulin resistance status for the whole cohort (*n*=109) at baseline sampling

	***Lactobacillus*** dominant ***n***/***N*** (%)	***Gardnerella*** dominant ***n***/***N*** (%)	High diversity ***n***/***N*** (%)	Total ***n***/***N*** (%)
**Obesity status**				
Non-obese (BMI <30.0 kg/m^2^)	41/67 (61.2)	16/67 (23.9)	10/67 (14.9)	67/67 (100)
Obese (≥30.0 kg/m^2^)	12/42 (28.6)	12/42 (28.6)	18/42 (42.8)	42/42 (100)
Total	53/109 (48.6)	28/109 (25.7)	28/109 (25.7)	109/109 (100)
*p* value^a^				**0.002**
**Diabetes status**				
Non-diabetic	49/97 (50.5)	25/97 (25.8)	23/97 (23.7)	97/97 (100)
Diabetic	4/12 (33.3)	3/12 (25.0)	5/12 (41.7)	12/12 (100)
Total	53/109 (48.6)	28/109 (25.7)	28/109 (25.7)	109/109 (100)
*P* value				0.451
**Insulin resistance status**^**b**^				
Non-insulin resistant	4/10 (40.0)	2/10 (20.0)	4/10 (40.0)	10/10 (100)
Insulin resistant	2/7 (28.6)	2/7 (28.6)	3/7 (42.9)	7/7 (100)
Total	6/17 (35.3)	4/17 (23.5)	7/17 (41.2)	17/17 (100)
*p* value				1.000

*BMI* body mass index

^a^A *p* value of less than 0.05 demonstrates a significant difference in the distribution of genus group present according to obesity, diabetic and insulin resistance status. Fisher’s exact test employed as small numbers were present for each group (e.g. diabetic)

^b^Where concomitant fasting serum samples were available, fasting glucose and fasting insulin levels were identified. Using these values, we were able to calculate the HOMA-IR, according to the formula: the product of fasting insulin (μU/L) multiplied by fasting glucose (nmol/L) divided by 22.5. The 2nd tertile was used as the cut-off to determine insulin resistance status. Insulin resistance cut-off value, 2.98

When women who had used antibiotics within 2 weeks of sample collection or had sexual intercourse within 48 h of sample collection were excluded, the prevalence of both the high diversity and *Lactobacillus*-dominant VMB remained significantly different in obese women in both categories (excluding intercourse, *p*=0.001; excluding recent antibiotic use, *p*=0.003) (Supplementary Table 3). When pre- and post-menopausal women were analysed separately, premenopausal obese women had a significantly higher prevalence of a high diversity VMB (*p*=0.001), even when oral contraceptive (OCP) users were excluded (*p*=0.003). There was a significant difference in the prevalence of each genus according to subcategories of normal weight, overweight and obese women (*p*<0.001) (Supplementary Table 3).

**Fig. 2:**
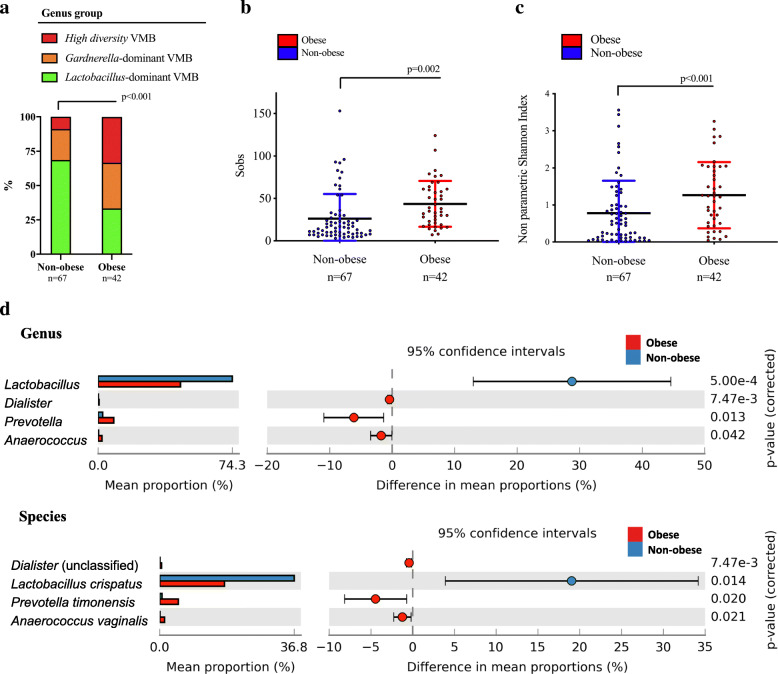
**a** The prevalence of the *Lactobacillus*-dominant genus group was lower in obese compared to non-obese women (*p*<0.001). **b** Number of species observed (Sobs) increased with obesity (*p*=0.002). **c** Significantly increased microbial diversity was seen among obese women (*p*=0006). **d** The vaginal microbiome of obese women was characterised at genus level by increased mean proportion (%) of *Dialister*, *Prevotella* and *Anaerococcus*. Results at species level showed increased mean proportion (%) of *Dialister* (unclassified), *Prevotella timonensis* and *Anaerococcus vaginalis* in obese women, and *Lactobacillus crispatus* dominated among non-obese women

Consistent with an increased prevalence of high diversity VMB (Fig. 2a), increased richness (number of species observed) and alpha diversity was observed in obese women (diversity, *p*= 0.006) (Fig. 2b, c, Supplementary Table 4). The vaginal microbiota of obese women was characterised by a greater mean proportion of anaerobic bacterial species, specifically unclassified *Dialister* spp. (unclassified) (*p*<0.001), *Anaerococcus vaginalis* (*p*=0.021) and *Prevotella timonensis* (*p*=0.020) (Fig. 2d).

To identify vaginal microbiota biomarkers specifically associated with obesity, we performed linear discriminant analysis (LDA) effect size (LefSe) modelling on the 16S rRNA gene sequence data collected from baseline samples (Supplementary Figure 1). Vaginal microbiota of obese women was enriched with members of *Bacteroidales* and *Clostridiales*, the *Prevotella* genus and the phylum *Actinobacteria*. Conversely, non-obese women were found to have enriched levels of *Lactobacillales* associated OTUs.

**Fig. 3:**
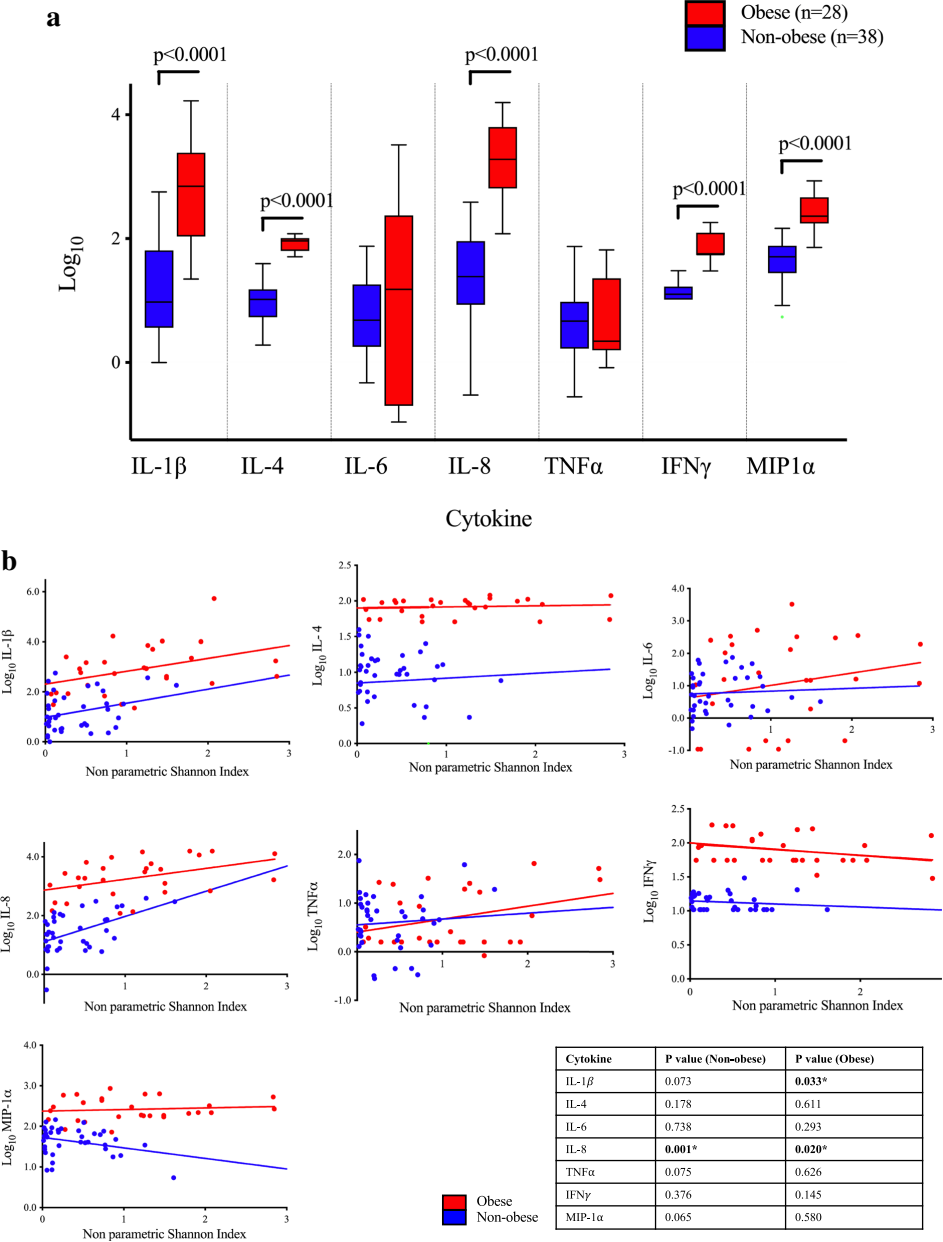
Pro-inflammatory local vaginal cytokine expression is increased in obesity. **a** Expression levels (log_10_) of seven cytokines measured among non-obese and obese women in the baseline cohort. Pro-inflammatory cytokines IL-1β, IL-8, IFNγ and MIP-1α all showed significantly increased expression among obese women (*p*<0.001). Anti-inflammatory cytokine IL-4 also had increased significance in obese women (*p*<0.001). **b** Expression of seven local cervicovaginal cytokine levels (log_10_) according to obesity status and species diversity (non-parametric Shannon indices). As the diversity of vaginal bacterial species increases, there is a significant increase in expression of pro-inflammatory cytokines IL-1β and IL-8, dependent on obesity status

Obese women had significantly increased expression of pro-inflammatory cytokines IL-1*β*, IL-6, IL-8, MIP-1*α*, IFN*γ* and TNF*α* compared to non-obese women (Fig. 3a, Supplementary Table 5). The anti-inflammatory cytokine IL-4 showed increased expression among obese women. As bacterial diversity increased among obese women (depicted using non-parametric Shannon Index), the expression of IL-1*β* and IL-8 but not the other cytokines, increased (Fig. 3b).

### Metabolic and vaginal microbiota compositional changes after bariatric surgery

At 6 months post bariatric surgery, the mean body weight of participants decreased on average by 19.2%, weight loss was similar for pre- and post-menopausal women (Supplementary Table 6). This change translated into a mean reduction of BMI of 10.3 (range 3.9 to 31.3) from a pre-surgery mean of 47.4 to a mean of 35.8 (6 months post-surgery). Five of 19 women moved from the obese category to the overweight category. The remaining 14 women persisted within the obese category.

**Fig. 4:**
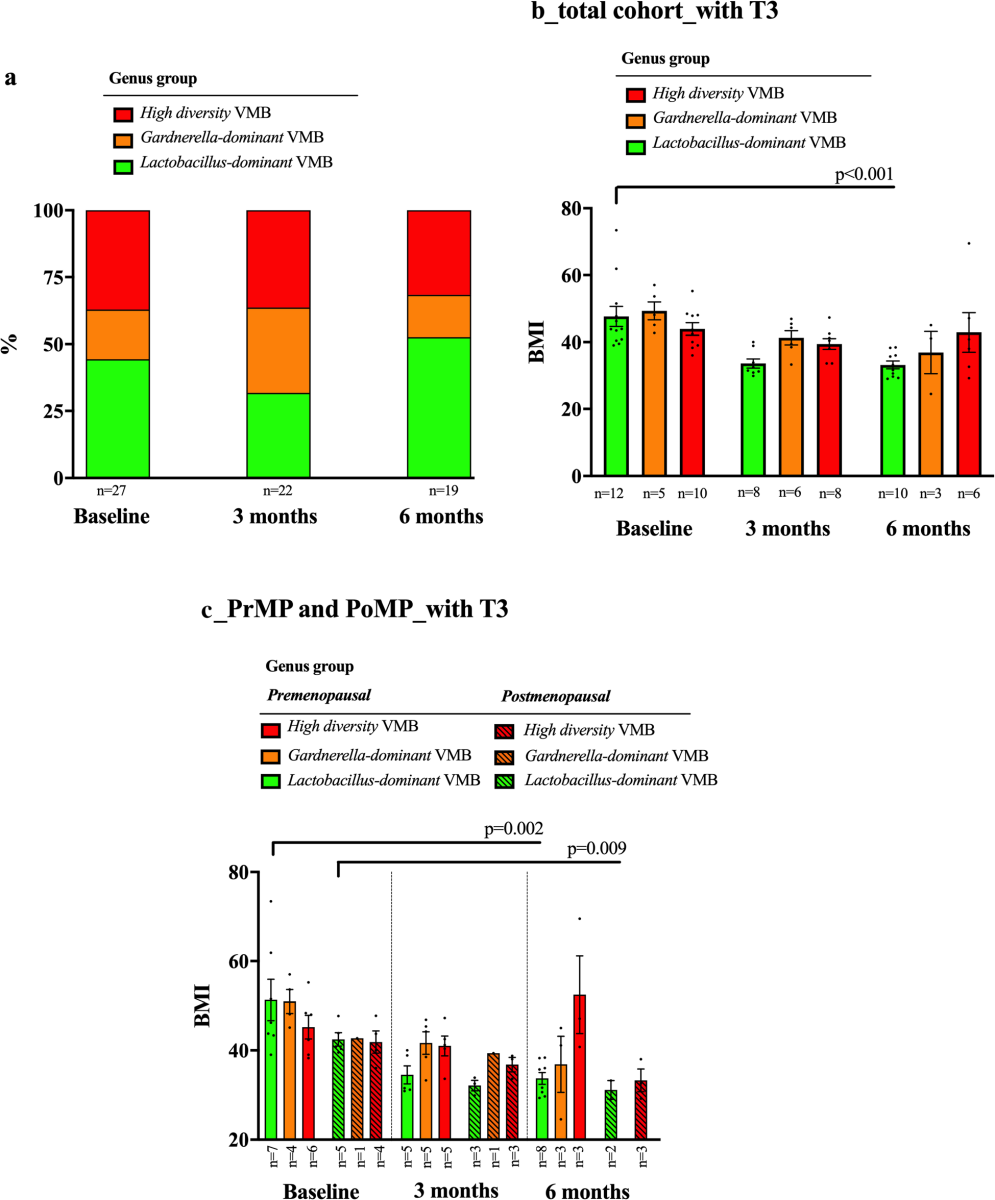
**a** Differences in proportion of vaginal microbiota groups from baseline (day of surgery) to 6 months post-surgery in the bariatric cohort. **b** The mean BMI of each of the three genus groups at baseline sampling and at 6 months post-surgery. There was a significant reduction in mean BMI in *Lactobacillus-*dominant VMB samples at 6 months post bariatric surgery (*p*<0.001). **c** The mean BMI of each of the three genus groups at baseline and 6 months post-surgery, according to menopause status. There was a significant reduction in mean BMI in the *Lactobacillus*-dominant group in premenopausal (*p*=0.002) and postmenopausal women (*p*=0.009)

In the subset of obese women undergoing bariatric surgery, there were no significant overall differences in VMB following surgery (Fig. 4a, Supplementary Figure 3). Neither were changes observed following bariatric surgery according to menopause status, diabetes or insulin resistance status (Table 3). Prior to surgery, there was no relationship between BMI and VMB structure; however, post-surgery women with *Lactobacillus*-dominant VMB had a significantly lower BMI than those with a high diversity VMB (Fig. 4b). This difference principally applied to pre-menopausal women (Fig. 4c). Local cervicovaginal cytokine levels in the bariatric surgery cohort at baseline sampling (*n*=27) and 6 months post-surgery (*n*=21) did not show any significant changes (Supplementary Figure 2).

**Table 3:** Prevalence of genus group present over serial sample collection timepoints in the total bariatric cohort and according to menopausal status, diabetes status and insulin resistance status

	***Lactobacillus*** dominant ***n***/***N*** (%)	***Gardnerella*** dominant ***n***/***N*** (%)	High diversity ***n***/***N*** (%)	Total ***n***/***N*** (%)
**Total bariatric surgery cohort**				
Baseline (day of surgery) (*n*=27)	10/27 (37.0)	5/27 (18.6)	12/27 (44.4)	27/27 (100)
3 months post-surgery (*n*=22)	7/22 (31.8)	6/22 (27.3)	9/22 (40.9)	22/22 (100)
6 months post-surgery (*n*=19)	10/19 (52.6)	2/19 (10.5)	7/19 (36.9)	19/19 (100)
Total (*n*=84)	31/84 (36.9)	16/84 (19.1)	37/84 (44.0)	84/84 (100)
*p* value^a^				0.506
**Pre-menopausal women only**				
Baseline (day of surgery) (*n*=17)	6/17 (35.3)	4/17 (23.5)	7/17 (41.2)	17/17 (100)
3 months post-surgery (*n*=15)	5/15 (33.3)	4/15 (26.7)	6/15 (40.0)	15/15 (100)
6 months post-surgery (*n*=14)	8/14 (57.1)	2/14 (14.3)	4/14 (28.6)	14/14 (100)
Total (*n*=58)	22/58 (37.9)	12/58 (20.7)	24/58 (41.4)	58/58 (100)
*p* value				0.572
**Post-menopausal women only**				
Baseline (day of surgery) (*n*=10)	4/10 (40.0)	1/10 (10.0)	5/10 (50.0)	10/10 (100)
3 months post-surgery (*n*=7)	2/7 (28.6)	2/7 (28.6)	3/7 (42.8)	7/7 (100)
6 months post-surgery (*n*=5)	2/5 (40.0)	0/5 (0)	3/5 (60.0)	5/5 (100)
Total (*n*=26)	9/26 (34.6)	4/26 (15.4)	13/26 (50.0)	26/26 (100)
*p* value				0.999
**Non-diabetic women only**				
Baseline (day of surgery) (*n*=19)	6/19 (31.6)	3/19 (15.8)	10/19 (52.6)	19/19 (100)
3 months post-surgery (*n*=16)	6/16 (37.5)	4/16 (25.0)	6/16 (37.5)	16/16 (100)
6 months post-surgery (*n*=14)	8/14 (57.1)	1/14 (7.2)	5/14 (35.7)	14/14 (100)
Total (*n*= 58)	21/58 (36.2)	9/58 (15.5)	28/58 (48.3)	58/58 (100)
*p* value				0.572
**Diabetic women only**				
Baseline (day of surgery) (*n*=8)	4/8 (50.0)	2/8 (25.0)	2/8 (25.0)	8/8 (100)
3 months post-surgery (*n*=6)	1/6 (16.7)	2/6 (33.3)	3/6 (50.0)	6/6 (100)
6 months post-surgery (*n*=5)	2/5 (40.0)	1/5 (20.0)	2/5 (40.0)	5/5 (100)
Total (*n*=26)	10/26 (38.5)	7/26 (26.9)	9/26 (34.6)	26/26 (100)
*p* value**				n/a
**Women without insulin resistance**^**c**^				
Baseline (day of surgery) (*n*=4)	1/4 (25.0)	1/4 (25.0)	2/4 (50.0)	4/4 (100)
3 months post-surgery (*n*=2)	0/2 (0)	0/2 (0)	2/2 (100)	2/2 (100)
6 months post-surgery (*n*=7)	4/7 (57.1)	1/7 (14.3)	2/7 (28.6)	7/7 (100)
Total (*n*=13)	5/13 (38.5)	2/13 (15.4)	6/13 (46.1)	13/13 (100)
*p* value				n/a
**Women with insulin resistance**				
Baseline (day of surgery) (*n*=5)	2/5 (40.0)	1/5 (20.0)	2/5 (40.0)	5/5 (100)
3 months post-surgery (*n*=1)	0/1 (0)	0/1 (0)	1/1 (100)	1/1 (100)
6 months post-surgery (*n*=1)	1/1 (100)	0/1 (0)	0/1 (0)	1/1 (100)
Total (*n*=7)	3/7 (42.9)	1/7 (14.3)	3/7 (42.9)	7/7 (100)
*p* value^b^				n/a

^a^A *p* value of less than 0.05 demonstrates a significant difference between the proportion (%) of genus group present (*Lactobacillus*-dominant, *Gardnerella*-dominant or high diversity VMB) over serial timepoints 0 to 6 months (McNemar’s Chi-square test)

^b^Sample size too small to compute *p* value using McNemar’s Chi-square test

^c^Where concomitant fasting serum samples were available, fasting glucose and fasting insulin levels were identified. Using these values, we were able to calculate the HOMA-IR, according to the following formula: the product of fasting insulin (μU/L) multiplied by fasting glucose (nmol/L) divided by 22.5. The 2nd tertile was used as the cut-off to determine insulin resistance status. Insulin resistance cut-off value: 2.98

Oestradiol levels and sex hormone-binding globulin (SHBG) levels were measured in a subset of 10 bariatric surgery patients at baseline and 6 months post-surgery (Supplementary Table 6). There were no overall differences in serum oestradiol levels in women pre- and post-surgery. There was no relationship between oestradiol levels and magnitude of weight loss 6 months post-surgery. Fasting glucose and insulin serum levels were also measured and HOMA-IR (insulin resistance index) was calculated. No associations were seen between oestradiol levels and SHBG levels or HOMA-IR and VMB group.

As oestrogen levels vary during the menstrual cycle and can be affected by exogenous sources of oestrogen such as OCP and hormone replacement therapy (HRT), we performed an analysis to include only postmenopausal women that were not taking HRT (Supplementary Table 6). There was no significant difference in the mean oestradiol levels (mean at baseline sampling was 55.0 pmol/L (range 37.0-82.0 pmol/L) and mean 6 months post-surgery was 37.0 pmol/L (range 37.0-37.0 pmol/L), (*p*=0.371)). SHBG levels increased in the total cohort after 6 months by 39.3% (*p*=0.008).

## Discussion

In the UK, two thirds of the female population is either overweight (30%), obese (27%) or morbidly obese (4%) [52]. Obesity has been associated with a multitude of adverse health outcomes in women [2, 4, 5, 12, 18], and although the mechanisms leading to these complications of obesity in women remain unclear, the vaginal microbiota composition may be important.

In our cohort, we found three VMB groups at genus level. Approximately half of the vaginal samples were categorised as *Lactobacillus*-dominant VMB, whilst the remaining samples were categorised in equal proportion as either *Gardnerella*-dominant (with a high relative abundance of *Gardnerella* spp. and low relative abundance of *Lactobacillus* spp.), or high diversity VMB (with a low relative abundance of each of *Lactobacillus* and *Gardnerella* spp., and increased bacterial diversity). When these samples were analysed with respect to obesity status, about 70% of obese women demonstrated a *Lactobacillus*-dominant VMB. This proportion is consistent with other reported studies. Brotman and colleagues showed that a *Lactobacillus*-dominant VMB is found in 80% of premenopausal women, but only 55% of postmenopausal women [53]. Our study represents a mixture of pre- and post-menopausal women. In those who were categorised as obese, 30% of women had a *Lactobacillus*-dominant VMB. This prevalence is lower than that found in non-obese postmenopausal women [53]. Our study shows that obesity associates with vaginal microbiota composition, with significantly higher vaginal bacterial species diversity and increased abundance of *Dialister*, *Prevotella* and *Anaerococcus* among obese women. Two previous studies, both from Korean patient cohorts, have described vaginal microbiota composition in relation to obesity in a non-pregnant population. The first study reported that obese women had a greater predominance of *Lactobacillus iners* compared to non-obese women who were more likely to have a *Lactobacillus crispatus*-dominant VMB [50]. However, this study was limited to the analysis of interrelationships between cervical *Lactobacillus* species only with non-*Lactobacillus* members of the microbiota not considered. Therefore, the generalisability of these findings are unclear [50]. Our findings lead us to conclude an increased prevalence of *Dialister* (unclassified), *Prevotella timonensis* and *Anaerococcus vaginalis* in obese women, and dominance of *Lactobacillus crispatus* among non-obese women (Fig. 2d). The second study, by Si et al., reported that obesity was associated with increased levels of *Prevotella*, and reduced *Lactobacillus* relative abundance consistent with our data [51].

The vaginal microbiota composition is dynamic and fluctuates throughout the life cycle with relative dominance of the niche by *Lactobacillus* spp. mediated by oestrogen-driven vaginal epithelium thickening and glycogen deposition, which is used as a primary energy source by lactic acid producing bacteria, encouraging a *Lactobacillus*-dominant VMB [54]. Accordingly, prepuberty and post-menopause vaginal microbiota composition associates with reduced glycogen levels [55] and a tendency towards a high diversity vaginal microbiota [56, 57]. In our study, vaginal microbiota composition in pre- and post-menopausal women was largely consistent with the published literature. In peri- or post-menopausal women with declining ovarian function, peripheral adipose tissue becomes the major source of production of unopposed circulating oestrogen by aromatisation of adrenal androstenedione to excess endogenous oestrogen [58–60]. In this group of women, reduced systemic oestrogen levels cause a decrease in glycogen deposition resulting in a vaginal epithelia that resembles pre-puberty with a thinner mucus layer and increased incidence of high diversity VMB [61, 62]. However, oestrogen levels are not higher in premenopausal obese women compared to non-obese. Freeman and co-workers found that premenopausal obese and overweight women had significantly lower oestradiol levels compared with non-obese women, independent of age, race or smoking [63]. Reduced circulating oestrogen concentrations are therefore a potential explanation for the lower prevalence of *Lactobacillus*-dominant VMB in obese women. It is also possible that due to restrictions in mobility caused by morbid obesity, female hygiene practices are affected. Local skin irritation and breakdown caused by the presence and rubbing of excess adipose tissue, together with persistent moisture may alter the local vaginal microbiota composition. The impact of women’s sexual, sanitary and hygiene practices such as douching on the vaginal microbiota is still controversial [64, 65].

Sustained weight loss may be brought about by significant lifestyle changes (diet and increased physical activity) or induced by bariatric surgery that can result in improved metabolic health. It has previously been reported that bariatric surgery can reduce cancer incidence [66], improve sex hormone profiles [67], polycystic ovarian syndrome symptoms [68], spontaneous and assisted conception rates [69–72] and reduce obstetric complications such as gestational diabetes, pregnancy-induced hypertension and macrosomia [73, 74]. Reversal or reduction in obesity brought about by bariatric surgery has been shown to be accompanied by metabolic improvement and reduction of alpha-diversity of the gut microbiota within 3 months post-operatively [75–77]. The impact of bariatric surgery on the vaginal microbiome has not however been previously assessed. In our study, in the subset of obese women undergoing bariatric surgery, there were no significant overall differences in VMB following surgery; however, 75% of these women remained in the obese range. Prior to surgery, there was no relationship between BMI and VMB structure; however, post-surgery women with *Lactobacillus*-dominant VMB had a significantly lower BMI than those with a high diversity VMB. Significant weight loss following bariatric surgery therefore associates with a tendency towards an “optimal” VMB. We did not, however, find any correlation between degree of weight loss and systemic oestradiol or SHBG concentrations. Additionally, SHBG increased in every woman at 6 months post-surgery which would lead to lower bioavailable oestrogen. It is therefore unlikely that any effect of bariatric surgery upon the VMB is principally due to changes in systemic oestradiol or SHBG concentrations.

In this study, we further explored how obesity may affect local inflammation and whether this is driven by increased diversity in the VMB. Previous studies have reported how a number of immune modulators in obesity (i.e. adipose tissue macrophages, cytokines and adipokines) can drive systemic inflammation [78, 79]. Other studies have shown that genital tract infection lead to changes in local immune factors that in turn have been linked to preterm birth [80] and bacterial vaginosis persistence [81]. Cervicovaginal cytokine levels (IL-1*β*, IL-6, IL-8, TNF*α*, MIP-1*α* and IFN*γ*) were increased in our obese population. This is in line with previous reports suggesting that modulation of the VMB in pregnancy increases local expression of each of these cytokines which correlates with increasing species diversity [82]. In the present study, however, there was an association between increasing bacterial species diversity and expression of only two pro-inflammatory cytokines IL-1*β* and IL-8. This data leads us to conclude that, in obesity, factors other than the VMB act to modulate cervicovaginal inflammation. We found no significant change in cervicovaginal cytokine levels following bariatric surgery, and cytokine concentrations were not different between the different genera groups. This finding again shows that much of the local cervicovaginal inflammation associated with obesity is unrelated to the vaginal microbiota.

Previous cross-sectional studies have highlighted that vaginal microbiota composition may be affected by obesity [50, 51]. A strength of the present study is the assessment of the vaginal microbiota composition in obese pre- and postmenopausal women separately, after exclusion of OCP and HRT-users and after taking into account confounding variables and hormonal serum levels. Furthermore, this is the first study to explore the temporal changes in the vaginal microbiota in a cohort of morbidly obese women undergoing surgically induced weight loss.

Although this study is one of the largest cohorts assessing the impact of obesity on the vaginal microbiota composition in women undergoing bariatric surgery, temporal data collected at each of the three timepoints was only available for a small number of patients which limits our ability to draw meaningful conclusions for many comparisons of interest. Recruitment of much larger numbers would be practically difficult. Follow-up for longer periods may show changes not revealed at 6 months, maximum weight loss is usually achieved 12-24 months after surgery [83, 84], although the majority of women who undergo bariatric surgery regain between 5 and 10% of their pre-operative weight by 2-3 years [84]. Furthermore, although negative controls have been included with each DNA extraction set, no amplicons were identified, and these were not subsequently sequenced. Given that vaginal samples are not considered to be of low biomass and we previously reported sequencing data of negative controls in similar contemporaneous patient cohorts [43, 85], we do not expect this to have led to spurious results. Consistent with this, we did not observe any common kit or environmental contaminants as being prominent features of any of the patient samples sequenced in this study.

## Conclusions

Obesity was found to be associated with higher vaginal microbiome diversity that may partly explain changes in local inflammation. Other factors beyond the VMB (i.e. endocrine) are likely to affect local inflammatory state. Surgery-induced weight loss did not change the VMB composition, although three quarters of the women remained obese 6 months post-surgery. Those with the greatest weight loss 6-month post-surgery were more likely to have a *Lactobacillus*-dominant VMB. A healthier VMB following bariatric surgery-induced weight loss may create a healthier local microenvironment to promote health, but since this does not associate with a reduction in local inflammation, other factors are likely to be involved and require further investigation.

## Materials and methods

### Study population—inclusion and exclusion criteria

We prospectively recruited non-pregnant women attending outpatient gynaecology and bariatric surgery clinics at Imperial College NHS Healthcare Trust between 2013 and 2016. A subset of this population was scheduled for bariatric surgery. Women were recruited irrespective of age, menopause status, ethnicity, parity, smoking, phase in menstrual cycle and contraception use. Women who were HIV, hepatitis B or C positive, had autoimmune disorders, or had a previous hysterectomy were excluded. Ethical approval was obtained from the National Research Ethics Service Committee London—Fulham (approval number 13/LO/0126) and the NHS West of Scotland Research Ethics Service Committee (WoSRES) (REC 14/WS/1098). All patients gave informed consent.

### Sample collection and processing

Cervicovaginal secretions were collected during the clinic visit from the posterior vaginal fornix with a BBL^TM^ CultureSwab^TM^ containing liquid Amies (Becton Dickinson, Oxford, UK) using a sterile, disposable speculum, without lubricant and immediately stored at −80 °C. A second transport microbiology swab (Transwab®) containing Amies gel medium was simultaneously collected for cytokine analysis. In the subset population of women planned for bariatric surgery (Gastric band, Roux-en-Y Gastric Bypass or Vertical Sleeve Gastrectomy), we collected serial vaginal swab samples on the day of surgery and at months 3 and 6 post-surgery (Supplementary Figure 4). Serial fasting blood samples were also collected on the day of surgery and at 6-month follow-up with the aim to correlate changes in vaginal microbiota to four serum markers known to be affected by surgery-induced weight loss and hyperinsulinaemia correction. Blood samples were centrifuged at 4472×*g* for 10 min and serum collected for freezing and storage in −80 °C.

A comprehensive interview and questionnaire were used to obtain all relevant gynaecological, medical and surgical history. Menopause status, type of contraception or HRT use and menstrual cycle phase (follicular or luteal) were documented. Ethnicity was self-reported as Caucasian, Asian, Black or other.

Whole-genomic bacterial DNA was extracted from the CultureSwab^TM^ using a QiAmp Mini DNA kit (Qiagen, Venlo, Netherlands) as described previously [36]. The second swab for cytokine analysis was thawed on ice and re-suspended in 350 μl phosphate-buffered saline solution with protease inhibitor (5 μl/ml; Sigma Aldrich). The suspension was centrifuged at 402×*g* for 2 min, and supernatant collected into a new 1.5 ml microcentrifuge tube. This centrifugation step was repeated to remove any remaining cellular debris. The cell-free supernatant was stored in −80 °C. Negative control swabs (blank, devoid of patient sample) were processed alongside each DNA extraction set. No amplicons were observed following PCR and gel electrophoresis of the negative controls, and these were not subsequently sequenced.

### Illumina MiSeq sequencing of 16S rRNA gene amplicons and data processing

The V1-V2 hypervariable regions of 16S rRNA genes were amplified by PCR using a forward and reverse fusion primer, described in detail in Supplementary Methods S1. Bacterial profiling using a MiSeq platform (Illumina, San Diego, CA, USA) was conducted at Research and Testing Laboratory (Lubbock, TX, USA). The 16S rRNA gene sequence data was analysed with bioinformatic software package Mothur [86] using the MiSeq SOP Pipeline. Sequence reads were quality checked and normalised to the lowest number of reads (*n*=1855) and singleton operational taxonomic units (OTUs); samples containing fewer than 10 reads were excluded. OTU taxonomies (from Phylum to Genus) were then determined using the RDP MultiClassifier script to generate the RDP taxonomy. Taxonomy level for species of the OTUs was determined using the USEARCH algorithm with 16S rRNA gene sequences from the cultured representatives from the RDP database [87]. Rare OTUs were defined as those present at less than 10 counts within the entire cohort. Alpha and beta diversity indices were calculated from these datasets with Mothur and R statistical package using the Vegan package.

### Serum biomarker and local vaginal cytokine analyses

Four serum markers including oestradiol (pmol/L), insulin (mIU/L), glucose (mmol/L) and sex hormone-binding globulin (SHBG, nmol/L) were quantified using ELISA at the Imperial College Healthcare NHS Trust North West London Pathology laboratory (Supplementary Methods S2).

Levels of interleukin (IL)-4, IL-6, IL-8, IL-1β, tumour necrosis factor-alpha (TNFα), interferon-gamma (IFN-γ) and macrophage inflammatory protein-1 alpha (MIP1α) in cell-free cervicovaginal secretion supernatants were determined using the Magnetic Luminex Screening Assay multiplex kit (R&D Systems, Minneapolis, MN, USA) on a MAGPIX Analyzer (Luminex® Corporation, s-Hertogenbosch, Netherlands), as per manufacturer’s instructions. Analytes were chosen based on evidence of inflammatory markers specific to adiposity [88–90].

### Statistical analyses

The population was categorised into two groups of interest for the main analysis, non-obese (BMI <30kg/m^2^) versus obese (BMI ≥30kg/m^2^). We performed further supplementary analyses to assess results for different obesity status subcategories and according to insulin resistance and diabetic status. The homeostatic model assessment for insulin resistance (HOMA-IR) was calculated by the following formula: the product of fasting insulin (μU/L) multiplied by fasting glucose (nmol/L) divided by 22.5 [91]. We used the 2nd tertile value of HOMA-IR as the cut-off to determine insulin resistance status (at 2.98). Differences in categorical clinical parameters between the two main groups of interest (non-obese versus obese) were assessed using Fisher’s exact test for each of the listed characteristics: age, ethnicity, parity, smoking, menopause, menstrual cycle, use of contraception, HRT use, diabetes status and treatment and abnormal high vaginal swab (HVS) results.

Significant differences between vaginal microbiota at genera taxonomic level were assessed using the Statistical Analysis of Metagenomic Profiles (STAMP) software package [92]. Dependent on genera hierarchical clustering analysis, *Lactobacillus* spp. or *Gardnerella* spp. abundance among selected phenotypic categories was investigated by assigning each patient sample into one of three groups (*Lactobacillus*-dominant, *Gardnerella*-dominant or high diversity VMB). Linear discriminant analysis (LDA) effect size (LEfSe) modelling was used to identify biomarkers based on obesity status, according to relative taxonomic abundance [93].

At genus taxonomic level, prevalence of each of the three categories relating to *Lactobacillus* or *Gardnerella* presence were compared between the two phenotypic categories (obese or non-obese) using Fisher’s exact test. We performed further sub-analyses for different weight categories, as well as by the presence of diabetes and/or insulin resistance status. A sensitivity analysis assessed whether the exclusion of women that had antibiotics less than 2 weeks before sample collection or those disclosing sexual intercourse within 48 h from sampling would affect the results. We further analysed the results for pre- and post-menopausal women separately and after exclusion of those taking oral hormonal contraception or hormone replacement therapy (HRT).

Expression levels of assayed cytokines were compared according to obesity status, prevalence of each genus group and diversity (non-parametric Shannon Index) at baseline sampling using two-way ANOVA. Where data fell outside the range of the standard curve for each analyte, either the minimal or maximal extrapolated or minimal or maximal value of the standard curve was used, where appropriate. Analyses were performed using Prism 8, *p* values <0.05 considered significant.

In the subset of women undergoing bariatric surgery, changes in serum markers introduced by weight loss were analysed. We further assessed the impact of surgically induced weight loss on the prevalence of each of the three genus groups at baseline, month 3 and 6 using McNemar’s Chi square test. We analysed the results for the full cohort and separately for pre- and post-menopausal women, and according to diabetic and insulin resistance status. Transition in vaginal microbiota across genus groups correlating with weight loss from baseline sampling to 6 months post-surgery was analysed for the total bariatric cohort and pre- and postmenopausal women separately. Cytokine and serum marker expression levels were compared between baseline sampling and 6 months after surgically induced weight loss.

## Supplementary Information

**Additional file 1: Supplementary Table 1.** Patient characteristics of the bariatric surgery cohort at baseline (*n*=27), and where indicated, for serial sample collection timepoints: 3 months post-surgery (*n*=22) and 6 months post-surgery (*n*=19). **Supplementary Table 2.** Prevalence of genus group according to BMI subcategories, diabetes and insulin resistance status in the total population at baseline sampling (*n*=109). **Supplementary Table 3.** Sensitivity analyses of the prevalence of each genus group present in the total population at baseline sampling according to obesity status: excluding women who had sexual intercourse less than 48 hours prior to sample collection; who had taken antibiotics within the 2 weeks prior to sample collection; premenopausal women only; premenopausal women excluding those taking OCP; postmenopausal women only; postmenopausal women excluding those taking HRT. **Supplementary Table 4.** Richness and Diversity Indices for whole patient cohort. **Supplementary Table 5.** Cytokine expression level according to obesity status of women in the total population, taken at baseline sampling. **Supplementary Table 6.** Summary of weight loss and serum marker changes from baseline (day of surgery) to 6-month follow-up in bariatric surgery cohort, with sensitivity analysis which excludes premenopausal women using the oral contraceptive pill and postmenopausal women using hormone replacement therapy. **Supplementary Figure 1.** Linear discriminant analysis effect size (LefSe) modelling identified vaginal microbiota biomarkers based on difference in obesity status, according to relative taxonomic abundance through all taxonomic levels. a) Cladogram representing taxa with different abundance according to obesity status in samples collected at baseline (*n*=109). The size of the circle is proportionate to the abundance of taxon present, yellow circles characterise non-significant differences in abundance at each taxonomic level, red/green coloured circles represent significant differences in abundance between non-obese and obese categories. b) Histogram of linear discriminant analysis (LDA) scores computed for features differentially abundant between non-obese and obese women. Relative abundance counts of *Actinobacteria, Bacteroidetes* and *Prevotella* were found to be significantly over-represented in obese women, whereas *Bacilli (Lactobacillales*) were enriched in non-obese samples (*n*=109, Welch’s t-test, LDA score greater than 2 used to determine discriminative features). *Key: LDA score; linear discriminant analysis score*. **Supplementary Figure 2.** Local cervicovaginal cytokine levels in the bariatric surgery cohort at baseline sampling (*n*=27) and 6 months post-surgery (*n*=21) did not show any significant changes. **Supplementary Figure 3.** Individual longitudinal profiling of *Lactobacillus* presence in the bariatric cohort (*n*=27) (P1 = patient number 1), according to menopause status. Each longitudinal sample was assigned to a genus group, either *Lactobacillus*-dominant vaginal microbiome (VMB), *Gardnerella*-dominant VMB, or high diversity VMB, as indicated by the colour-coded rectangle. **Supplementary Figure 4.** Data collection protocol for participants undergoing bariatric surgery. **S1.** Illumina MiSeq sequencing of 16S rRNA gene amplicons and data processing. **S2.** Quantification of serum markers using ELISA.

## Data Availability

Public access to sequence data and accompanying metadata can be obtained at the European Nucleotide Archive’s (ENA) Sequence Read Archive (SRA) (accession number: PRJEB40616).
