# Supplementary material for: The association between obesity and weight loss after bariatric surgery on the vaginal microbiota
Source: Microbiome. 2021 May 28;9:124. doi: 10.1186/s40168-021-01011-2 (PMC8164250; doi:10.1186/s40168-021-01011-2)
Supplement: Supplementary file 2 — Additional file 1: Supplementary Table 1. Patient characteristics of the bariatric surgery cohort at baseline (n=27), and where indicated, for serial sample collection timepoints: 3 months post-surgery (n=22) and 6 months post-surgery (n=19). Supplementary Table 2. Prevalence of genus group according to BMI subcategories, diabetes and insulin resistance status in the total population at baseline sampling (n=109). Supplementary Table 3. Sensitivity analyses of the prevalence of each genus group present in the total population at baseline sampling according to obesity status: excluding women who had sexual intercourse less than 48 hours prior to sample collection; who had taken antibiotics within the 2 weeks prior to sample collection; premenopausal women only; premenopausal women excluding those taking OCP; postmenopausal women only; postmenopausal women excluding those taking HRT. Supplementary Table 4. Richness and Diversity Indices for whole patient cohort. Supplementary Table 5. Cytokine expression level according to obesity status of women in the total population, taken at baseline sampling. Supplementary Table 6. Summary of weight loss and serum marker changes from baseline (day of surgery) to 6-month follow-up in bariatric surgery cohort, with sensitivity analysis which excludes premenopausal women using the oral contraceptive pill and postmenopausal women using hormone replacement therapy. Supplementary Figure 1. Linear discriminant analysis effect size (LefSe) modelling identified vaginal microbiota biomarkers based on difference in obesity status, according to relative taxonomic abundance through all taxonomic levels. a) Cladogram representing taxa with different abundance according to obesity status in samples collected at baseline (n=109). The size of the circle is proportionate to the abundance of taxon present, yellow circles characterise non-significant differences in abundance at each taxonomic level, red/green coloured circles represent significant d [file 40168_2021_1011_MOESM2_ESM.docx]

**Supplementary Materials:**

**Supplementary tables**

**Supplementary Table 1.** Patient characteristics of the bariatric surgery cohort at baseline (n=27), and where indicated, for serial sample collection timepoints: 3 months post-surgery (n=22) and 6 months post-surgery (n=19).

|  | ***Day of surgery***  **(n=27)** | ****3 months***  ***post-surgery***  **(n=22)** | ****6 months***  ***post-surgery***  **(n=19)** |
| --- | --- | --- | --- |
| **Age (years)** |  |  |  |
| Mean (SD, range) | 48 (8.91, 28-65) | n/a | n/a |
| **Ethnicity, n/N (%)** |  |  |  |
| Caucasian | 18/27 (66.7) | n/a | n/a |
| Asian | 3/27 (11.1) | n/a | n/a |
| Black | 6/27 (22.2) | n/a | n/a |
| Other | 0/27 (0) | n/a | n/a |
| *Total (n=27)* | 27/27 (100) | n/a | n/a |
| **Parity, n/N (%)** |  |  |  |
| Nulliparous | 3/27 (11.1) | n/a | n/a |
| Parous | 24/27 (88.9) | n/a | n/a |
| *Total (n=27)* | 27/27 (100) | n/a | n/a |
| **Smoking status, n/N (%)** |  |  |  |
| Current smoker | 1/27 (3.7) | n/a | n/a |
| Non-smoker | 26/27 (96.3) | n/a | n/a |
| *Total (n=27)* | 27/27 (100) | n/a | n/a |
| **BMI distribution, n/N (%)** |  |  |  |
| BMI (kg/m^2^), mean (range) | 46.5 (36.0-73.4) | 39.1 (29.9-47.7) | 38.9 (24.5-69.5) |
| Normal (18.5-25.0kg/m^2^) | n/a | n/a | n/a |
| Overweight (25.1-30.0kg/m^2^) | n/a | 1/22 (4.5) | 5/19 (26.3) |
| Obese (≥30.0kg/m^2^) | 27/27 (100) | 21/22 (95.5) | 14/19 (73.7) |
| *Total* | 27/27 (100) | 22/22 (100) | 19/19 (100) |
| **HVS results, n/N (%)** |  |  |  |
| Normal | 22/27 (81.5) | 18/22 (81.8) | 19/19 (100) |
| Abnormal | 5/27 (18.5) | 4/22 (18.2) | 0/19 (0) |
| *Total* | 27/27 (100) | 22/22 (100) | 19/19 (100) |
| **Abnormal HVS results, n/N (%)** |  |  |  |
| *Bacterial vaginosis* | 0/5 (0) | 0/4 (0) | 0 |
| *E.coli* | 0/5 (0) | 0/4 (0) | 0 |
| *S.aureus* | 1/5 (20.0) | 0/4 (0) | 0 |
| *Group B streptococcus* | 2/5 (40.0) | 2/4 (50.0) | 0 |
| *Yeast* | 1/5 (20.0) | 1/4 (25.0) | 0 |
| *Mixed coliforms* | 1/5 (20.0) | 0/4 (0) | 0 |
| *S. milleri* | 0/5 (0) | 1/4 (25.0) | 0 |
| *Total* | 5/5 (100) | 4/4 (100) | 0 |
| **Menopause status, n/N (%)** |  |  |  |
| Premenopausal | 17/27 (63.0) | n/a | n/a |
| Postmenopausal | 10/27 (37.0) | n/a | n/a |
| *Total* | 27/27 (100) | n/a | n/a |
| **Phase of menstrual cycle (PrMP), n/N (%)** | |  |  |
| Luteal | 6/17 (35.3) | 8/15 (53.3) | 2/13 (15.4) |
| Follicular | 8/17 (47.0) | 5/15 (33.3) | 9/13 (69.2) |
| Ovulation | 1/17 (5.9) | 0/15 (0) | 0/13 (0) |
| Unknown | 2/17 (11.8) | 2/15 (13.3) | 2/13 (15.4) |
| *Total* | 17/17 (100) | 15/15 (100) | 13/13 (100) |
| **Use of contraception (PrMP women only), n/N (%)** | |  |  |
| Nil | 13/17 (76.5) | 12/15 (80.0) | 12/14 (85.7) |
| Condoms | 0/17 (0) | 0/15 (0) | 0/14 (0) |
| COCP | 0/17 (0) | 0/15 (0) | 0/14 (0) |
| POP | 0/17 (0) | 0/15 (0) | 0/14 (0) |
| Copper IUD | 0/17 (0) | 0/15 (0) | 0/14 (0) |
| Mirena IUS | 4/17 (23.5) | 3/15 (20.0) | 2/14 (14.3) |
| Vaginal ring | 0/17 (0) | 0/15 (0) | 0/14 (0) |
| Contraceptive implant | 0/17 (0) | 0/15 (0) | 0/14 (0) |
| Contraceptive injection | 0/17 (0) | 0/15 (0) | 0/14 (0) |
| *Total* | 17/17 (100) | 15/15 (100) | 14/14 (100) |
| **Use of HRT (PoMP), n/N (%)** | |  |  |
| Yes | 1/10 (10.0) | 1/7 (14.3) | 1/6 (16.7) |
| No | 9/10 (90.0) | 6/7 (85.7) | 5/6 (83.3) |
| *Total* | 10/10 (100) | 7/7 (100) | 6/6 (100) |
| **Diabetes status, n/N (%)** |  |  |  |
| Non-diabetic | 19/27 (70.4) | 16/22 (72.7) | 14/19 (73.7) |
| Diabetic | 8/27 (29.6) | 6/22 (27.3) | 5/19 (26.3) |
| *Total* | 27/27 (100) | 22/22 (100) | 19/19 (100) |
| **Diabetic treatment, n/N (%)** |  |  |  |
| Diet control only | 1/8 (12.5) | 1/6 (16.7) | 1/5 (20.0) |
| Metformin alone | 3/8 (37.5) | 3/6 (50.0) | 2/5 (40.0) |
| Metformin & 2nd diabetic medication | 0/8 (0) | 0/6 (0) | 0/5 (0) |
| Other oral antiglycaemic medication | 0/8 (0) | 0/6 (0) | 0/5 (0) |
| Insulin (alone or with oral medication) | 4/8 (50.0) | 2/6 (33.3) | 2/5 (40.0) |
| *Total* | 8/8 (100) | 6/6 (100) | 5/5 (100) |
| **HOMA-IR, n/N (%)**** |  |  |  |
| Insulin resistant | 4/9 (44.4) | n/a | 1/8 (12.5) |
| Non-insulin resistant | 5/9 (55.6) | n/a | 7/8 (87.5) |
| *Total* | 9/9 (100) | n/a | 8/8 (100) |

*None of the women used vaginal pessaries during or immediately prior to any of the serial visits. Only one woman used antibiotics within two weeks from sample collection and two women had sexual intercourse within 48 hours from sample collection at 3 months. At baseline sample collection, none of the participants had taken antibiotics within the past two weeks, used pessaries or had sexual intercourse within the 48 hours preceding vaginal swab sample collection.

** HOMA-IR was calculated according to the formula: the product of fasting insulin (μU/L) and fasting glucose (nmol/L) divided by 22.5. The 2nd tertile was used as the cut-off to determine insulin resistance status. Insulin resistance cut-off value: 2.98.

*BMI, body mass index; COCP, combined oral contraceptive pill; E.coli, escherichia coli; HOMA-IR, homeostatic model of assessment - insulin resistance; HRT, hormone replacement therapy; HVS, high vaginal swab; IUD, intrauterine device; IUS, intrauterine system; PoMP, postmenopausal; POP, progesterone-only pill; PrMP, premenopausal; S.aureus, staphylococcus aureus; SD, standard deviation; S.milleri, streptococcus milleri.*

**Supplementary Table 2.** Prevalence of genus group according to BMI subcategories, diabetes and insulin resistance status in the total population at baseline sampling (n=109).

|  | ***Lactobacillus* dominant**  **n/N (%)** | ***Gardnerella* dominant**  **n/N (%)** | **High diversity**  **n/N (%)** | **TOTAL**  **n/N (%)** |
| --- | --- | --- | --- | --- |
| BMI CATEGORY | |  |  |  |
| Normal weight (18.5-24.9kg/m^2^) | 25/33 (75.8) | 4/33 (12.1) | 4/33 (12.1) | 33/33 (100.0) |
| Overweight (25.0-29.9kg/m^2^) | 27/34 (79.4) | 6/34 (17.6) | 1/34 (2.9) | 34/34 (100.0) |
| Obese (≥30.0kg/m^2^) | 14/42 (33.3) | 14/42 (33.3) | 14/42 (33.3) | 42/42 (100.0) |
| Total | 66/109 (60.6) | 24/109 (22.0) | 19/109 (17.4) | 109/109 (100.0) |
| p value* |  |  |  | **0.0001** |
| BMI CATEGORY | |  |  |  |
| Normal weight (18.5-24.9kg/m^2^) | 25/33 (75.8) | 4/33 (12.1) | 4/33 (12.1) | 33/33 (100.0) |
| Overweight & Obese (≥25.0kg/m^2^) | 41/76 (54.0) | 20/76 (26.3) | 15/76 (19.7) | 76/76 (100.0) |
| Total | 66/109 (60.6) | 24/109 (22.0) | 19/109 (17.4) | 109/109 (100.0) |
| p value |  |  |  | **0.096** |
| BMI CATEGORY | | |  |  |
| Non-obese (BMI <30.0kg/m^2^) | 51/67 (76.1) | 11/67 (16.4) | 5/67 (7.5) | 67/67 (100.0) |
| Obese (30.0-34.9kg/m^2^) | 1/8 (12.5) | 4/8 (50.0) | 3/8 (37.5) | 8/8 (100.0) |
| Morbidly obese (≥35.0kg/m^2^) | 13/34 (38.2) | 10/34 (29.4) | 11/34 (32.4) | 34/34 (100.0) |
| Total | 65/109 (59.7) | 25/109 (22.9) | 19/109 (17.4) | 109/109 (100.0) |
| p value |  |  |  | **0.0001** |
| BMI CATEGORY AND DIABETIC STATUS | |  |  |  |
| Non-obese (BMI <30.0kg/m^2^) & non-diabetic | 50/65 (76.9) | 11/65 (16.9) | 4/65 (6.2) | 65/65 (100.0) |
| Non-obese (BMI <30.0kg/m^2^) & diabetic | 1/2 (50.0) | 0/2 (0) | 1/2 (50.0) | 2/2 (100.0) |
| Obese (≥30.0kg/m^2^) & non-diabetic | 10/32 (31.2) | 11/32 (34.4) | 11/32 (34.4) | 32/32 (100.0) |
| Obese (≥30.0kg/m^2^) & diabetic | 4/10 (40.0) | 3/10 (30.0) | 3/10 (30.0) | 10/10 (100.0) |
| Total | 65/109 (59.7) | 25/109 (22.9) | 19/109 (17.4) | 109/109 (100.0) |
| p value |  |  |  | **0.001** |
| BMI CATEGORY AND INSULIN RESISTANCE STATUS** | | |  |  |
| Non-obese (BMI <30.0kg/m^2^) & non-insulin resistant | 3/5 (60.0) | 2/5 (40.0) | 0/5 (0) | 5/5 (100.0) |
| Non-obese (BMI <30.0kg/m^2^) & insulin resistant | 0/2 (0) | 1/2 (50.0) | 1/2 (50.0) | 2/2 (100.0) |
| Obese (≥30.0kg/m^2^) & non-insulin resistant | 1/5 (20.0) | 1/5 (20.0) | 3/5 (60.0) | 5/5 (100.0) |
| Obese (≥30.0kg/m^2^) & insulin resistant | 2/5 (40.0) | 1/5 (20.0) | 2/5 (40.0) | 5/5 (100.0) |
| Total | 6/17 (35.3) | 5/17 (29.4) | 6/17 (35.3) | 17/17 (100.0) |
| p value |  |  |  | 0.482 |

*A p-value of less than 0.05 demonstrates that there is a significant difference in the proportion (%) of each genus group according to BMI category, BMI category and diabetic status, or BMI category and insulin resistance status (Fisher exact test).

** Where concomitant fasting serum samples were available, fasting glucose and fasting insulin levels were identified. Using these values, we were able to calculate the HOMA-IR, according to the formula: the product of fasting insulin (μU/L) multiplied by fasting glucose (nmol/L) divided by 22.5. The 2nd tertile was used as the cut-off to determine insulin resistance status. Insulin resistance cut-off value: 2.98.

*BMI, body mass index.*

**Supplementary Table 3.** Sensitivity analyses of the prevalence of each genus group present in the total population at baseline sampling according to obesity status: excluding women who had sexual intercourse less than 48 hours prior to sample collection; who had taken antibiotics within the 2 weeks prior to sample collection; premenopausal women only; premenopausal women excluding those taking OCP; postmenopausal women only; postmenopausal women excluding those taking HRT.

|  | ***Lactobacillus* dominant**  **n/N (%)** | ***Gardnerella* dominant**  **n/N (%)** | **High diversity**  **n/N (%)** | **TOTAL**  **n/N (%)** |
| --- | --- | --- | --- | --- |
| **BMI CATEGORY**  **excluding intercourse <48hr (n=104)** |  |  |  |  |
| Non-obese (BMI <30.0kg/m^2^) | 46/62 (74.2) | 11/62 (17.7) | 5/62 (8.1) | 62/62 (100) |
| Obese (≥30.0 kg/m^2^) | 14/42 (33.3) | 14/42 (33.3) | 14/42 (33.4) | 42/42 (100) |
| Total (n=104) | 60/104 (57.7) | 24/104 (24.0) | 2/104 (18.3) | 104/104 (100) |
| p value* |  |  |  | **0.001** |
| **BMI CATEGORY**  **excluding antibiotic use last two weeks (n=107)** | |  |  |  |
| Non-obese (BMI <30.0 kg/m^2^) | 49/65 (75.4) | 11/65 (16.9) | 15/65 (23.1) | 65/65 (100) |
| Obese (≥30.0 kg/m^2^) | 14/42 (33.3) | 14/42 (33.3) | 14/42 (33.4) | 42/42 (100) |
| Total (n=107) | 63/107 (58.9) | 25/107 (23.4) | 29/107 (27.1) | 107/107 (100) |
| p value |  |  |  | **0.003** |
| **BMI CATEGORY**  **Premenopausal (n=76)** |  |  |  |  |
| Non-obese (BMI <30.0 kg/m^2^) | 45/50 (90.0) | 5/50 (10.0) | 0/50 (0) | 50/50 (100) |
| Obese (≥30.0 kg/m^2^) | 9/26 (34.6) | 10/26 (38.5) | 7/26 (26.9) | 26/26 (100) |
| Total (n=76) | 54/76 (71.1) | 15/76 (19.7) | 7/76 (9.2) | 76/76 (100) |
| p value |  |  |  | **0.001** |
| **BMI CATEGORY**  **Premenopausal, excluding OCP users (n=47)** | |  |  |  |
| Non-obese (BMI <30.0 kg/m^2^) | 23/27 (85.2) | 4/27 (14.8) | 0/27 (0) | 27/27 (100.0) |
| Obese (≥30.0 kg/m^2^) | 8/20 (40.0) | 8/20 (40.0) | 4/20 (20.0) | 20/20 (100.0) |
| Total (n=47) | 31/47 (66.0) | 12/47 (25.5) | 4/47 (8.5) | 47/47 (100.0) |
| p value |  |  |  | **0.003** |
| **BMI CATEGORY:**  **Postmenopausal (n=33)** |  |  |  |  |
| Non-obese (BMI <30.0 kg/m^2^) | 6/17 (35.3) | 6/17 (35.3) | 5/17 (29.4) | 17/17 (100.0) |
| Obese (≥30.0 kg/m^2^) | 5/16 (31.3) | 4/16 (25.0) | 7/16 (43.7) | 16/16 (100.0) |
| Total (n=33) | 11/33 (33.3) | 10/33 (30.3) | 12/33 (36.4) | 33/33 (100.0) |
| p value |  |  |  | 0.672 |
| **BMI CATEGORY:**  **Postmenopausal, excluding HRT users (n=27)** | |  |  |  |
| Non-obese (BMI <30.0 kg/m^2^) | 5/12 (41.7) | 4/12 (33.3) | 3/12 (25.0) | 12/12 (100.0) |
| Obese (≥30.0 kg/m^2^) | 4/15 (26.7) | 4/15 (26.7) | 7/15 (46.7) | 15/15 (100.0) |
| Total (n=27) | 9/27 (33.3) | 8/27 (29.6) | 10/27 (37.0) | 27/27 (100.0) |
| p value |  |  |  | 0.498 |

***** A p-value of less than 0.05 demonstrates that there is a significant difference between the proportion of each genus group according to BMI category (Fisher exact test).

*BMI, body mass index; CST, community-state type; HRT, hormone replacement therapy; OCP, oral contraceptive pill; SD, standard deviation.*

**Supplementary Table 4.** Richness and Diversity Indices for whole patient cohort.

| **Sample ID** | **BMI category** | **BMI (kg/m ^2^)** | **Number of sequences** | **Species observed** | **Inverse Simpson** | **InvSimpson_Ici** | **InvSimpson_hci** | **Non-parametric Shannon** | **Coverage*** |
| --- | --- | --- | --- | --- | --- | --- | --- | --- | --- |
| R25 | Obese | 30.9 | 1855 | 107 | 14.055787 | 13.176711 | 15.060542 | 3.253872 | 0.97035 |
| R26 | Obese | 30.0 | 1855 | 57 | 4.300597 | 4.080022 | 4.546384 | 2.037968 | 0.983827 |
| R1 | Obese | 33.0 | 1855 | 28 | 1.616492 | 1.550429 | 1.688435 | 0.913827 | 0.989757 |
| R2 | Non-obese | 28.0 | 1855 | 22 | 1.358325 | 1.313293 | 1.406554 | 0.633298 | 0.992992 |
| R3 | Non-obese | 22.0 | 1855 | 96 | 12.025322 | 11.19328 | 12.990996 | 3.122685 | 0.975741 |
| R30 | Non-obese | 29.0 | 1855 | 24 | 1.738312 | 1.676634 | 1.8047 | 0.832354 | 0.989757 |
| R31 | Non-obese | 27.0 | 1855 | 36 | 1.073742 | 1.055644 | 1.092471 | 0.337059 | 0.983288 |
| R32 | Obese | 50.0 | 1855 | 124 | 2.916632 | 2.764716 | 3.086214 | 2.041035 | 0.951482 |
| R27 | Non-obese | 27.0 | 1855 | 54 | 3.608593 | 3.416503 | 3.823571 | 1.876281 | 0.98221 |
| R6 | Non-obese | 27.0 | 1855 | 74 | 6.468009 | 6.028521 | 6.976616 | 2.573156 | 0.984906 |
| R33 | Non-obese | 28.0 | 1855 | 81 | 2.265268 | 2.166794 | 2.373118 | 1.503104 | 0.977898 |
| R28 | Obese | 41.0 | 1855 | 21 | 1.021923 | 1.012358 | 1.031671 | 0.149162 | 0.989218 |
| R34 | Non-obese | 28.0 | 1855 | 65 | 7.078695 | 6.55384 | 7.694934 | 2.655709 | 0.983827 |
| R101 | Obese | 37.5 | 1855 | 76 | 8.867588 | 8.292466 | 9.52843 | 2.847481 | 0.981671 |
| R76 | Obese | 46.0 | 1855 | 69 | 6.314041 | 5.838281 | 6.87422 | 2.67178 | 0.987601 |
| R102 | Non-obese | 23.7 | 1855 | 83 | 19.448799 | 18.200763 | 20.880592 | 3.439697 | 0.983827 |
| R103 | Non-obese | 27.2 | 1855 | 54 | 2.472483 | 2.349668 | 2.608845 | 1.48891 | 0.981132 |
| R104 | Non-obese | 24.2 | 1855 | 30 | 2.776834 | 2.671506 | 2.890808 | 1.335552 | 0.991375 |
| R96 | Non-obese | 26.9 | 1855 | 21 | 1.07333 | 1.055434 | 1.091843 | 0.256812 | 0.990836 |
| R105 | Non-obese | 20.1 | 1855 | 6 | 1.005414 | 1.000706 | 1.010167 | 0.037429 | 0.997305 |
| R106 | Non-obese | 27.5 | 1855 | 30 | 2.483794 | 2.359088 | 2.62242 | 1.424461 | 0.99407 |
| R97 | Non-obese | 19.6 | 1855 | 17 | 1.053785 | 1.038502 | 1.069524 | 0.2068 | 0.996765 |
| R107 | Obese | 37.3 | 1855 | 69 | 3.634395 | 3.441534 | 3.850155 | 2.036755 | 0.980593 |
| R98 | Non-obese | 23.4 | 1855 | 92 | 5.602659 | 5.298355 | 5.944048 | 2.419825 | 0.967116 |
| R99 | Non-obese | 19.5 | 1855 | 27 | 1.051468 | 1.036538 | 1.066835 | 0.24853 | 0.988679 |
| R108 | Non-obese | 24.1 | 1855 | 23 | 2.114494 | 2.04272 | 2.191496 | 0.986785 | 0.991914 |
| R100 | Non-obese | 21.5 | 1855 | 66 | 4.35808 | 4.186474 | 4.544355 | 2.001493 | 0.98221 |
| R109 | Obese | 30.8 | 1855 | 12 | 1.103685 | 1.081955 | 1.126305 | 0.28318 | 0.997305 |
| R118 | Non-obese | 25.6 | 1855 | 93 | 1.563022 | 1.496258 | 1.636022 | 1.362946 | 0.971429 |
| R115 | Non-obese | 24.8 | 1855 | 61 | 3.539289 | 3.388152 | 3.704541 | 1.796822 | 0.982749 |
| R110 | Non-obese | 24.9 | 1855 | 10 | 1.009777 | 1.003436 | 1.016199 | 0.067306 | 0.995148 |
| R116 | Non-obese | 23.8 | 1855 | 19 | 2.332131 | 2.27589 | 2.391221 | 1.009714 | 0.991914 |
| R114 | Obese | 33.7 | 1855 | 64 | 4.943524 | 4.705114 | 5.207384 | 2.171628 | 0.982749 |
| R124 | Obese | 36.1 | 1855 | 23 | 1.398403 | 1.352704 | 1.447297 | 0.628446 | 0.990836 |
| R117 | Non-obese | 26.0 | 1855 | 9 | 1.027423 | 1.01674 | 1.038332 | 0.106767 | 0.996226 |
| R119 | Obese | 35.7 | 1855 | 79 | 12.484826 | 11.732251 | 13.340568 | 3.040841 | 0.980593 |
| R122 | Obese | 32.1 | 1855 | 60 | 2.186071 | 2.112803 | 2.264602 | 1.246436 | 0.980593 |
| R121 | Obese | 34.0 | 1855 | 30 | 3.924542 | 3.763993 | 4.099397 | 1.686634 | 0.991914 |
| R29 | Non-obese | 22.0 | 1855 | 153 | 12.071838 | 11.081854 | 13.256051 | 3.559739 | 0.969811 |
| R9 | Obese | 39.5 | 1855 | 19 | 1.019699 | 1.010645 | 1.028916 | 0.134312 | 0.990296 |
| R13 | Obese | 47.7 | 1855 | 29 | 1.533776 | 1.481298 | 1.590108 | 0.729675 | 0.987062 |
| R23 | Obese | 40.8 | 1855 | 49 | 2.039465 | 1.970247 | 2.113723 | 1.095176 | 0.980593 |
| R21 | Obese | 39.0 | 1855 | 25 | 1.262777 | 1.22706 | 1.300635 | 0.501851 | 0.989218 |
| R11 | Obese | 47.8 | 1855 | 30 | 1.132762 | 1.107678 | 1.159008 | 0.421707 | 0.988679 |
| R4 | Obese | 43.9 | 1855 | 14 | 1.015271 | 1.007321 | 1.023346 | 0.09922 | 0.993531 |
| R20 | Obese | 36.0 | 1855 | 77 | 3.040204 | 2.847186 | 3.261296 | 2.050877 | 0.977358 |
| R43 | Obese | 43.8 | 1855 | 33 | 1.043407 | 1.029761 | 1.05742 | 0.257834 | 0.983288 |
| R72 | Obese | 39.0 | 1855 | 17 | 1.757839 | 1.705025 | 1.81403 | 0.729308 | 0.995148 |
| R41 | Obese | 40.0 | 1855 | 83 | 8.084822 | 7.4747 | 8.803399 | 2.834241 | 0.983288 |
| R45 | Obese | 46.2 | 1855 | 32 | 1.732344 | 1.674275 | 1.794585 | 0.832615 | 0.986523 |
| R56 | Obese | 53.6 | 1855 | 39 | 2.125343 | 2.026938 | 2.233789 | 1.261524 | 0.98814 |
| R19 | Obese | 55.2 | 1855 | 49 | 2.448892 | 2.336817 | 2.572258 | 1.439922 | 0.985445 |
| R95 | Obese | 45.1 | 1855 | 61 | 3.51626 | 3.323515 | 3.732738 | 1.91488 | 0.983288 |
| R22 | Obese | 73.4 | 1855 | 8 | 1.015254 | 1.007322 | 1.023313 | 0.072599 | 0.996765 |
| R24 | Obese | 43.4 | 1855 | 53 | 1.416809 | 1.36468 | 1.473078 | 0.936913 | 0.978976 |
| R53 | Obese | 40.4 | 1855 | 15 | 1.256633 | 1.221843 | 1.293461 | 0.435815 | 0.993531 |
| R81 | Obese | 43.7 | 1855 | 35 | 2.345885 | 2.25569 | 2.443593 | 1.213548 | 0.987601 |
| R38 | Obese | 48.4 | 1855 | 38 | 2.571711 | 2.481601 | 2.668612 | 1.241839 | 0.983827 |
| R68 | Obese | 42.4 | 1855 | 51 | 3.118286 | 2.933434 | 3.328002 | 1.797428 | 0.985984 |
| R75 | Obese | 57.0 | 1855 | 58 | 4.719325 | 4.496937 | 4.964852 | 2.076005 | 0.985445 |
| R17 | Obese | 48.1 | 1855 | 43 | 1.913495 | 1.820303 | 2.016743 | 1.321634 | 0.991375 |
| R50 | Obese | 38.3 | 1855 | 19 | 1.32863 | 1.288655 | 1.371165 | 0.520466 | 0.991914 |
| R89 | Obese | 48.0 | 1855 | 34 | 3.135571 | 3.013133 | 3.268382 | 1.490834 | 0.989757 |
| R48 | Obese | 61.9 | 1855 | 16 | 1.104047 | 1.082538 | 1.126428 | 0.279503 | 0.99407 |
| R90 | Obese | 42.7 | 1855 | 16 | 1.908199 | 1.844275 | 1.976714 | 0.850693 | 0.993531 |
| R77 | Obese | 51.5 | 1855 | 61 | 2.452601 | 2.343095 | 2.572845 | 1.490677 | 0.977898 |
| A18 | Non-obese | 28.0 | 1855 | 9 | 2.03001 | 1.98831 | 2.073497 | 0.803021 | 0.997305 |
| A21 | Non-obese | 28.0 | 1855 | 20 | 1.557667 | 1.497824 | 1.622491 | 0.769553 | 0.991375 |
| A22 | Non-obese | 22.0 | 1855 | 16 | 1.834651 | 1.759159 | 1.916912 | 0.901189 | 0.994609 |
| A24 | Non-obese | 22.0 | 1855 | 12 | 1.218193 | 1.184867 | 1.253447 | 0.445476 | 0.996765 |
| A26 | Non-obese | 27.0 | 1855 | 13 | 1.028622 | 1.017655 | 1.039828 | 0.126478 | 0.996765 |
| M138 | Non-obese | 24.0 | 1855 | 23 | 1.429571 | 1.382315 | 1.480172 | 0.63984 | 0.99407 |
| M139 | Non-obese | 27.0 | 1855 | 33 | 1.243094 | 1.207258 | 1.281122 | 0.593572 | 0.991914 |
| M140 | Non-obese | 26.0 | 1855 | 11 | 1.349518 | 1.307961 | 1.393801 | 0.50919 | 0.995687 |
| M141 | Non-obese | 24.0 | 1855 | 17 | 1.258627 | 1.22265 | 1.296786 | 0.483622 | 0.99407 |
| M142 | Non-obese | 26.0 | 1855 | 16 | 1.420111 | 1.375252 | 1.467995 | 0.560355 | 0.99407 |
| M143 | Non-obese | 27.0 | 1855 | 18 | 2.010331 | 1.958907 | 2.064528 | 0.867825 | 0.992992 |
| M144 | Non-obese | 26.0 | 1855 | 6 | 1.005414 | 1.000706 | 1.010167 | 0.037429 | 0.997305 |
| M145 | Non-obese | 27.0 | 1855 | 7 | 1.006502 | 1.00134 | 1.011718 | 0.044904 | 0.996765 |
| M147 | Non-obese | 29.0 | 1855 | 10 | 2.243791 | 2.177046 | 2.314758 | 0.9681 | 0.996765 |
| M153 | Non-obese | 27.0 | 1855 | 11 | 1.048856 | 1.034462 | 1.063656 | 0.161159 | 0.995687 |
| M163 | Non-obese | 28.0 | 1855 | 10 | 1.029672 | 1.01854 | 1.04105 | 0.116323 | 0.996226 |
| M165 | Non-obese | 29.0 | 1855 | 12 | 1.092019 | 1.071901 | 1.112907 | 0.242396 | 0.996226 |
| M166 | Non-obese | 27.0 | 1855 | 24 | 1.435958 | 1.384878 | 1.490949 | 0.710587 | 0.989218 |
| M167 | Non-obese | 29.0 | 1855 | 15 | 2.12991 | 2.056657 | 2.208574 | 0.961867 | 0.995687 |
| M178 | Non-obese | 29.0 | 1855 | 31 | 3.013675 | 2.855009 | 3.191015 | 1.612788 | 0.993531 |
| M179 | Non-obese | 23.0 | 1855 | 7 | 1.108096 | 1.086378 | 1.130701 | 0.231475 | 0.997844 |
| M47 | Non-obese | 27.0 | 1855 | 11 | 1.070614 | 1.05321 | 1.088603 | 0.198161 | 0.995148 |
| M58 | Non-obese | 23.0 | 1855 | 21 | 1.044485 | 1.030692 | 1.058651 | 0.203423 | 0.990836 |
| M76 | Non-obese | 27.0 | 1855 | 8 | 1.034153 | 1.022188 | 1.046401 | 0.117877 | 0.997305 |
| M77 | Obese | 31.0 | 1855 | 7 | 1.006502 | 1.00134 | 1.011718 | 0.044904 | 0.996765 |
| M86 | Non-obese | 29.0 | 1855 | 15 | 1.024148 | 1.014099 | 1.034398 | 0.12401 | 0.995148 |
| MK222 | Non-obese | 23.0 | 1855 | 7 | 1.339059 | 1.296013 | 1.385063 | 0.525366 | 0.998383 |
| MK229 | Non-obese | 23.0 | 1855 | 4 | 1.003243 | 0.999602 | 1.006911 | 0.022469 | 0.998383 |
| MK242 | Non-obese | 25.0 | 1855 | 8 | 1.009775 | 1.003437 | 1.016195 | 0.057417 | 0.996765 |
| MK253 | Non-obese | 25.0 | 1855 | 7 | 1.288086 | 1.249776 | 1.32882 | 0.471486 | 0.999461 |
| MK254 | Non-obese | 27.0 | 1855 | 8 | 1.009776 | 1.003437 | 1.016196 | 0.056577 | 0.997305 |
| MK276 | Non-obese | 24.0 | 1855 | 5 | 1.004328 | 1.00012 | 1.008571 | 0.029951 | 0.997844 |
| MK277 | Non-obese | 22.0 | 1855 | 5 | 1.004328 | 1.00012 | 1.008571 | 0.029951 | 0.997844 |
| MK284 | Non-obese | 26.0 | 1855 | 3 | 1.00216 | 0.99919 | 1.005149 | 0.014983 | 0.998922 |
| MK290 | Non-obese | 24.0 | 1855 | 4 | 1.003243 | 0.999602 | 1.006911 | 0.022469 | 0.998383 |
| MK291 | Non-obese | 28.0 | 1855 | 14 | 1.84875 | 1.794331 | 1.906573 | 0.775593 | 0.995687 |
| MK292 | Non-obese | 23.0 | 1855 | 7 | 1.006502 | 1.00134 | 1.011718 | 0.044904 | 0.996765 |
| MK293 | Non-obese | 24.0 | 1855 | 5 | 1.00868 | 1.002713 | 1.014718 | 0.040188 | 0.998922 |
| MK297 | Non-obese | 25.0 | 1855 | 6 | 1.005414 | 1.000706 | 1.010167 | 0.037429 | 0.997305 |
| MK299 | Non-obese | 22.0 | 1855 | 10 | 2.002209 | 1.975834 | 2.029297 | 0.754044 | 0.997305 |
| MK300 | Non-obese | 23.0 | 1855 | 9 | 1.070773 | 1.05329 | 1.088847 | 0.193226 | 0.997305 |
| MK301 | Non-obese | 23.0 | 1855 | 21 | 2.04051 | 1.936563 | 2.156249 | 1.259717 | 0.995687 |
| MK302 | Non-obese | 24.0 | 1855 | 5 | 1.035172 | 1.023078 | 1.047555 | 0.102833 | 0.998383 |

*BMI, body mass index*

**Good’s coverage index provides a score for each sample analysed to estimate what percent of the total species is represented in the sample (where Coverage is defined as 1 – ((number of singleton OTUs) / (number of reads in the sample)).*

**Supplementary Table 5.** Cytokine expression level according to obesity status of women in the total population, taken at baseline sampling.

| **Cytokine** | **N** | **Non-obese**  (median ± IQR) | **N** | **Obese**  (median ± IQR) | **P value*** |
| --- | --- | --- | --- | --- | --- |
| **IL-1**$\boldsymbol{\beta}$ | 38 | 9.51 (3.89-55.02) | 28 | 585.50 (91.48-2248.13) | <0.0001 |
| **IL-4** | 38 | 10.50 (5.77-14.91) | 28 | 90.42 (60.52-101.19) | <0.0001 |
| **IL-6** | 38 | 4.83 (1.92-17.83) | 28 | 41.08 (13.32-274.59) | 0.0006 |
| **IL-8** | 38 | 24.42 (8.67-85.12) | 28 | 1901.35 (608.00-6063.16) | <0.0001 |
| **IFN**$\boldsymbol{\gamma}$ | 38 | 16.34 (14.20-17.90) | 28 | 112.90 (91.65-158.60) | <0.0001 |
| **MIP-1**$\boldsymbol{\alpha}$ | 38 | 51.17 (28.72-75.05) | 28 | 230.82 (180.00-445.75) | <0.0001 |
| **TNF**$\boldsymbol{\alpha}$ | 38 | 4.68 (1.82-9.15) | 28 | - 1. (2.84-26.16) | 0.0361 |

* A p-value of less than 0.05 demonstrates that there is a significant difference in cytokine expression level according to obesity status (obese versus non-obese) (unpaired t-test).

*BMI, body mass index; IL, interleukin; IQR, interquartile range, NA, not available.*

**Supplementary Table 6.** Summary of weight loss and serum marker changes from baseline (day of surgery) to 6-month follow-up in bariatric surgery cohort, with sensitivity analysis which excludes premenopausal women using the oral contraceptive pill and postmenopausal women using hormone replacement therapy.

|  | **N** | **Baseline** | **N** | **6 months**  **post-surgery** | **p value*** | **q value** |
| --- | --- | --- | --- | --- | --- | --- |
| **Absolute body weight (kg), mean (range)** | | |  |  |  |  |
| Combined (all samples) | 19 | 125.7 (92.0-222.1) | 19 | 101.54 (63.6-187.9) | **3.81^e-06^** | NA |
| Premenopausal only | 14 | 127.8 (92.0-222.1) | 14 | 105.2 (63.6-187.9) | **1.22^e-04^** | NA |
| Postmenopausal only | 5 | 112.20 (96.0-148.0) | 5 | 88.7 (68.0-111.0) | 0.063 | NA |
| **Body weight loss (% mean)** |  |  |  |  |  |  |
| Combined (all samples) | - | - | 19 | -19.2% | - |  |
| Premenopausal only | - | - | 14 | -17.7% | - |  |
| Postmenopausal only | - | - | 5 | -20.9% | - |  |
| **Serum levels, total cohort**  **mean (range)** | | |  |  |  |  |
| Oestradiol (pmol/L) | 10 | 132.8 (37.0-370.0) | 10 | 236.0 (37.0-894.0) | 0.343 | 0.441 |
| Fasting glucose (mmol/L) | 10 | 5.6 (3.8-9.5) | 10 | 5.0 (4.0-6.7) | 0.102 | 0.190 |
| Fasting insulin (mIU/L) | 10 | 29.0 (8.5-79.0) | 10 | 14.2 (5.3-35.0) | 0.105 | 0.190 |
| SHBG (nmol/L) | 10 | 39.5 (11.0-75.0) | 10 | 65.1 (32.0-104.0) | **0.008** | 0.036 |
| **Serum levels, premenopausal only****  **mean (range)** | | |  |  |  |  |
| Oestradiol (pmol/L) | 6 | 187.7 (82.0-370.0) | 6 | 368.2 (90.0-894.0) | 0.156 | 0.394 |
| Fasting glucose (mmol/L) | 6 | 5.7 (3.8-9.5) | 6 | 5.0 (4.0-6.7) | 0.207 | 0.394 |
| Fasting insulin (mIU/L) | 6 | 33.0 (11.7-79.0) | 6 | 18.2 (5.5-35.0) | 0.438 | 0.563 |
| SHBG (nmol/L) | 6 | 45.4 (14.3-75.0) | 6 | 66.8 (32.0-104.0) | **0.063** | 0.281 |
| **Serum levels, postmenopausal only**  **mean (range)** | | |  |  |  |  |
| Oestradiol (pmol/L) | 4 | 50.5 (37.0-82.0) | 4 | 37.8 (37.0-40.0) | 0.423 | 0.543 |
| Fasting glucose (mmol/L) | 4 | 5.4 (4.7-5.8) | 4 | 4.9 (4.3-6.5) | 0.357 | 0.536 |
| Fasting insulin (mIU/L) | 4 | 23.0 (8.5-44.0) | 4 | 8.2 (5.3-13.4) | 0.125 | 0.281 |
| SHBG (nmol/L) | 4 | 30.8 (11.0-58.0) | 4 | 62.5 (40.0-101.0) | 0.125 | 0.281 |
| **Serum levels, postmenopausal only**  **excluding HRT, mean (range)** | | |  |  |  |  |
| Oestradiol (pmol/L) | 3 | 55.0 (37.0-82.0) | 3 | 37.0 (37.0-37.0) | 0.371 | 0.643 |
| Fasting glucose (mmol/L) | 3 | 5.4 (4.7-5.8) | 3 | 5.1 (4.4-6.5) | 0.750 | 0.844 |
| Fasting insulin (mIU/L) | 3 | 16.0 (8.5-24.0) | 3 | 6.5 (5.3-7.6) | 0.250 | 0.563 |
| SHBG (nmol/L) | 3 | 37.3 (23.0-58.0) | 3 | 70.0 (41.0-101.0) | 0.250 | 0.563 |

*A p-value of less than 0.05 demonstrates that there is a significant difference between the characteristic of interest at baseline (day of surgery) and month 6 post-surgery (paired Wilcoxon sum rank test).

**A sensitivity analysis excluding OCP users among premenopausal women was also considered, however none of the premenopausal women with matched pre- and post-surgery samples were OCP users.

*CRP, c-reactive protein; IGF-1, insulin-like growth factor-1; IGFBP-3, insulin-growth factor binding protein-3; SHBG, sex hormone binding globulin.*

**Supplementary Figures**

**a b**


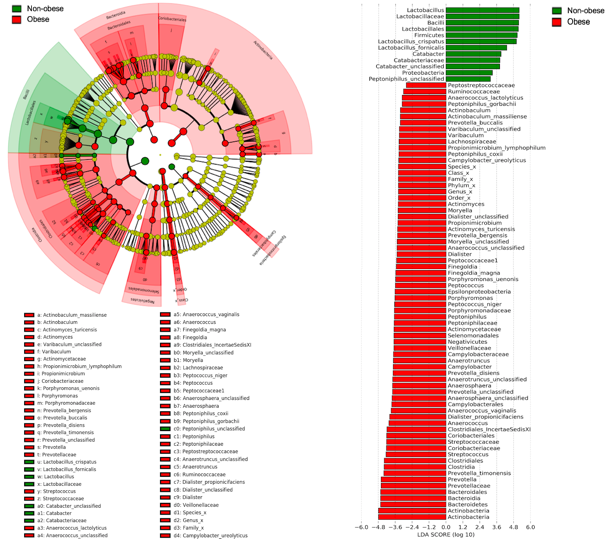


**Supplementary Figure 1**. Linear discriminant analysis effect size (LefSe) modelling identified vaginal microbiota biomarkers based on difference in obesity status, according to relative taxonomic abundance through all taxonomic levels. **a)** Cladogram representing taxa with different abundance according to obesity status in samples collected at baseline (n=109). The size of the circle is proportionate to the abundance of taxon present, yellow circles characterise non-significant differences in abundance at each taxonomic level, red/green coloured circles represent significant differences in abundance between non-obese and obese categories. **b)** Histogram of linear discriminant analysis (LDA) scores computed for features differentially abundant between non-obese and obese women. Relative abundance counts of *Actinobacteria, Bacteroidetes* and *Prevotella* were found to be significantly over-represented in obese women, whereas *Bacilli (Lactobacillales*) were enriched in non-obese samples (n=109, Welch’s t-test, LDA score greater than 2 used to determine discriminative features). *Key: LDA score; linear discriminant analysis score.*

**
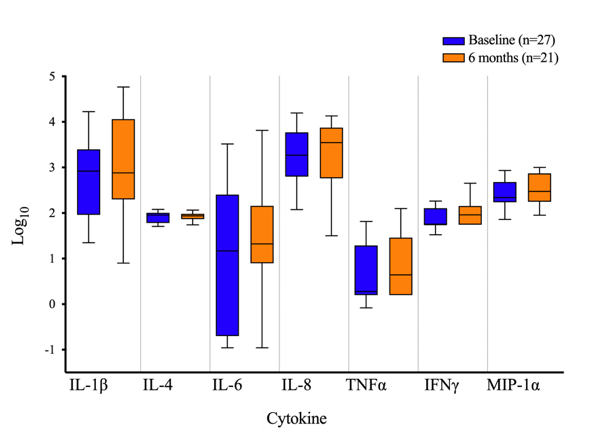
**

**Supplementary Figure 2.** Local cervicovaginal cytokine levels in the bariatric surgery cohort at baseline sampling (n=27) and 6 months post-surgery (n=21) did not show any significant changes.

**Timepoint at sampling**

|  | Baseline |  | 3 months |  | 6 months |  |  |
| --- | --- | --- | --- | --- | --- | --- | --- |
| P9 |  | > |  | > |  |  | *Gardnerella-*dominant VMB |
| P11 |  | > |  | > |  |  |  |
| P16 |  | > |  | > |  |  |  |
| P25 |  | > |  | > |  |  |  |
| P8 |  | > |  | > |  |  |  |
| P15 |  | > | > | > |  |  |  |
| P22 |  | > |  | > |  |  |  |
| P14 |  | > |  | > |  |  |  |
| P12 |  | > |  | > |  |  |  |
| P21 |  | > |  | > |  |  |  |
| P19 |  | > |  | > |  |  |  |
| P23 |  | > |  | > |  |  |  |
| P27 |  | > |  | > | > |  |  |
| P4 |  | > |  | > | > |  |  |
| P13 |  | > |  | > |  |  |  |
| P20 |  | > | > | > | > |  |  |
| P24 |  | > |  | > |  |  |  |
|  |  |  |  |  |  |  |  |
|  |  |  |  |  |  |  |  |
| P17 |  | > |  | > | > |  |  |
| P3 |  | > |  | > |  |  |  |
| P1 |  | > |  | > |  |  |  |
| P6 |  | > |  | > |  |  |  |
| P26 |  | > |  | > | > |  |  |
| P10 |  | > |  | > |  |  |  |
| P2 |  | > | > | > | > |  |  |
| P5 |  | > |  | > |  |  |  |
| P7 |  | > | > | > | > |  |  |
| P18 |  | > | > | > | > |  |  |

*Lactobacillus*-dominant VMB

Premenopausal

High diversity VMB

Postmenopausal

**Supplementary Figure 3.** Individual longitudinal profiling of *Lactobacillus* presence in the bariatric cohort (n=27) (P1 = patient number 1), according to menopause status. Each longitudinal sample was assigned to a genus group, either *Lactobacillus*-dominant vaginal microbiome (VMB), *Gardnerella*-dominant VMB, or high diversity VMB, as indicated by the colour-coded rectangle.

**
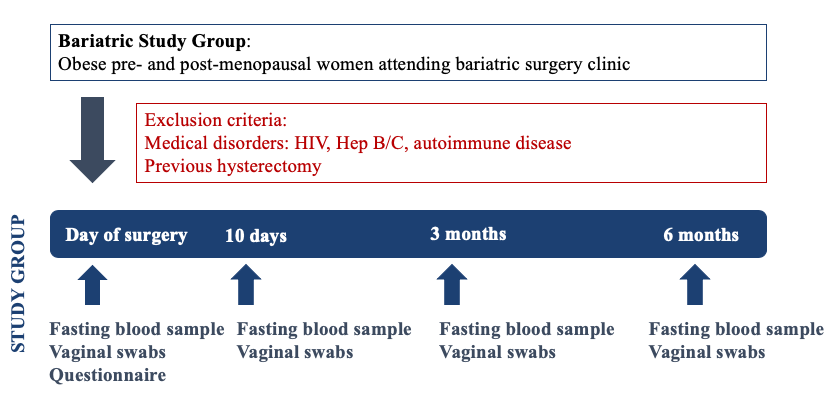
**

**Supplementary Figure 4.** Data collection protocol for participants undergoing bariatric surgery.

**Supplementary Methods**

**S1. Illumina MiSeq sequencing of 16S rRNA gene amplicons and data processing**

The V1-V2 hypervariable regions of 16S rRNA genes were amplified by PCR using mixed Forward primer sets as follows:

| 28F-YM GAGTTTGATYMTGGCTCAG |  |
| --- | --- |
| 28F-Borrellia GAGTTTGATCCTGGCTTAG | |
| 28F-Chloroflex GAATTTGATCTTGGTTCAG | |
| 28F-Bifdo GGGTTCGATTCTGGCTCAG | |

These primers are mixed at a 4:1:1:1 ratio (28F-YM is at 4 parts) with 388R reverse primers. Bacterial profiling using a MiSeq platform (Illumina, San Diego, CA, USA) was conducted at Research and Testing Laboratory (Lubbock, TX, USA).

**S2. Quantification of serum markers using ELISA**

Fasting serum samples were used to four analyse serum markers including oestradiol (pmol/L), insulin (mIU/L), glucose (mmol/L) and sex hormone binding globulin (SHBG, nmol/L). Oestradiol, glucose and insulin were analysed using ci8000SR Abbott Architect System (Abbott Diagnostics, Ireland). SHBG was analysed using Immulite 2000XPi System (Siemens, Germany). All assays were run in duplicate according to manufacturer’s instructions, and laboratory-specific reference ranges.

Population reference intervals, Imperial College Healthcare NHS Trust North West London Pathology Laboratory.

| **Fasting serum** | **Reference interval (units)** |
| --- | --- |
| **Glucose** *Adult female* | 3.0 – 6.0 (mmol/L) |
| **Insulin** | 3-15 mIU/L |
| **Oestradiol** *Female early follicular*  *Pre-ovulatory*  *Luteal*  *Post-menopause* | 200-500 pmol/L  500-1,500 pmol/l  250-1,000 pmol/L  <100 pmol/L |
| **SHBG** | 30-100 nmol/L |

*CRP, c-reactive protein; IGFBP3, insulin-like growth factor binding protein-3; IGF-1, insulin-like growth factor-1; HMW, high molecular weight; SHBG, sex hormone binding globulin; yrs, years***.**
